# An engineering strategy to target activated EGFR with CAR T cells

**DOI:** 10.1016/j.crmeth.2024.100728

**Published:** 2024-03-15

**Authors:** Markus Dobersberger, Delia Sumesgutner, Charlotte U. Zajc, Benjamin Salzer, Elisabeth Laurent, Dominik Emminger, Elise Sylvander, Elisabeth Lehner, Magdalena Teufl, Jacqueline Seigner, Madhusudhan Reddy Bobbili, Renate Kunert, Manfred Lehner, Michael W. Traxlmayr

**Affiliations:** 1 Department of Chemistry, Institute of Biochemistry, BOKU University, 1190 Vienna, Austria; 2 CD Laboratory for Next Generation CAR T Cells, 1090 Vienna, Austria; 3 St. Anna Children’s Cancer Research Institute, CCRI, 1090 Vienna, Austria; 4 BOKU Core Facility Biomolecular & Cellular Analysis, BOKU University, 1190 Vienna, Austria; 5 Department of Biotechnology, Institute of Animal Cell Technology and Systems Biology, BOKU University, 1190 Vienna, Austria; 6 Department of Biotechnology, Institute of Molecular Biotechnology, BOKU University, 1190 Vienna, Austria; 7 Ludwig Boltzmann Institute for Traumatology, Research Center in Cooperation with AUVA, 1200 Vienna, Austria; 8 St. Anna Children’s Hospital, Department of Pediatrics, Medical University of Vienna, 1090 Vienna, Austria

**Keywords:** protein engineering strategy, yeast surface display technology, activated EGFR, EGF, TGF-α, CAR T cells, Nur77 reporter cell line, antigen-binding domain, protein-protein interaction, conformational specificity

## Abstract

Chimeric antigen receptor (CAR) T cells have shown remarkable response rates in hematological malignancies. In contrast, CAR T cell treatment of solid tumors is associated with several challenges, in particular the expression of most tumor-associated antigens at lower levels in vital organs, resulting in on-target/off-tumor toxicities. Thus, innovative approaches to improve the tumor specificity of CAR T cells are urgently needed. Based on the observation that many human solid tumors activate epidermal growth factor receptor (EGFR) on their surface through secretion of EGFR ligands, we developed an engineering strategy for CAR-binding domains specifically directed against the ligand-activated conformation of EGFR. We show, in several experimental systems, that the generated binding domains indeed enable CAR T cells to distinguish between active and inactive EGFR. We anticipate that this engineering concept will be an important step forward to improve the tumor specificity of CAR T cells directed against EGFR-positive solid cancers.

## Highlights

• Human cancers frequently show constitutive EGFR activation
• We present an engineering strategy to target these activated receptor conformations
• Engineered binding domains specifically recognize ligand-activated EGFR
• These binders enable specific targeting of activated EGFR with CAR T cells

## Motivation

A key challenge in the CAR T cell field is the lack of truly tumor-specific antigens. Most tumor antigens are expressed to some extent in healthy tissues, causing undesired on-target/off-tumor toxicities when being targeted with CAR T cells. Therefore, methods to improve the tumor specificity of CAR T cells are urgently needed. EGFR represents a prominent example of a well-established tumor-associated antigen that is broadly expressed at low levels in healthy tissues. Here, we developed a protein engineering strategy to generate EGFR-specific CARs with improved tumor specificity.

## Graphical abstract

**Figure:**
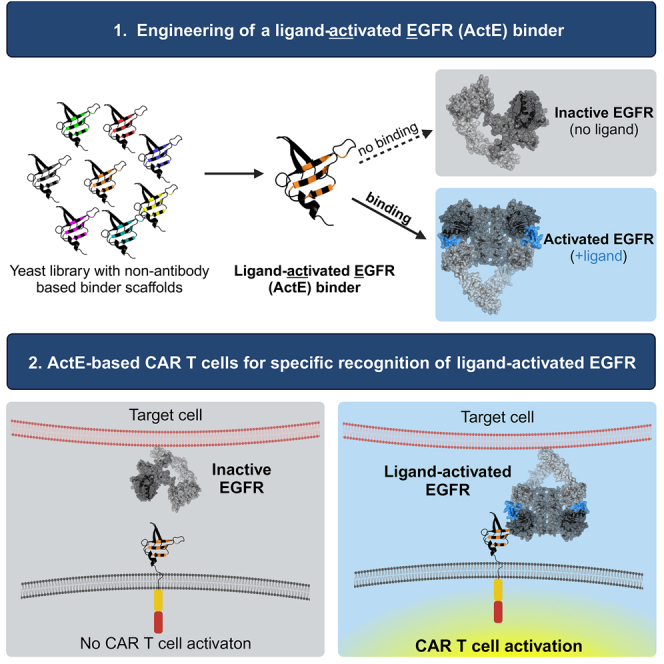

## Teaser

EGFR is often found to be constitutively activated in human cancer. Dobersberger et al. develop an engineering strategy for the generation of binding domains specifically recognizing ligand-activated EGFR. Incorporation of these binders into CAR molecules allows for specific targeting of the activated state of EGFR with CAR T cells.

## Introduction

The epidermal growth factor receptor (EGFR) is a receptor tyrosine kinase that is frequently overexpressed and/or activated in a variety of human cancers, including glioblastoma, non-small cell lung cancer, and breast cancer, among others.^1^^,^^2^^,^^3^ Due to its prominent role in tumorigenesis, a wide range of EGFR-targeting drugs have been clinically approved. These can be largely grouped into two main categories: (1) monoclonal antibodies (mAbs) targeting the extracellular domain of EGFR (e.g., cetuximab, panitumumab, and necitumumab) and (2) tyrosine kinase inhibitors (TKIs), which block the intracellular kinase domain of EGFR (e.g., erlotinib, gefitinib, and osimertinib).^4^^,^^5^^,^^6^

More recently, T cells expressing chimeric antigen receptors (CAR T cells) have emerged as another promising therapeutic class of cancer treatments, in particular for hematologic malignancies.^7^^,^^8^ In addition, CAR T cell approaches are also extensively studied in the context of solid tumors, for which EGFR is a potential target antigen. Multiple laboratories have investigated EGFR-targeting CAR T cells in preclinical and clinical studies, demonstrating detectable but limited clinical efficacy.^9^^,^^10^^,^^11^^,^^12^^,^^13^^,^^14^ Thus, EGFR-targeting CAR T cell therapies with improved potency are highly desired.

However, EGFR is known to be broadly expressed in multiple organs in the human body.^1^^,^^15^ As a consequence, it is well established that EGFR-targeting therapies lead to severe on-target/off-tumor toxicities; i.e., side effects due to recognition of EGFR in healthy tissues.^15^^,^^16^^,^^17^ This is reflected in the emergence of similar toxicities (most frequently skin rashes) upon treatment with EGFR-directed mAbs and TKIs, respectively, despite highly dissimilar mechanisms of action of these two drug classes.^15^^,^^16^^,^^17^ Of note, despite limited clinical efficacy of EGFR-targeting CAR T cells in early clinical trials, these side effects, including skin rashes, have been observed as well,^11^^,^^12^ suggesting that an increased potency will inevitably result in more severe toxicities due to enhanced reactivity against EGFR-positive healthy tissues. In this regard, it is worth mentioning that CAR T cells directed against the closely related antigen ERBB2 have caused fatal toxicities due to recognition of low levels of ERBB2 on lung epithelial cells.^18^ On-target/off-tumor toxicities due to recognition of low-level antigen expression in healthy tissues are also known for bispecific T cell engagers (BiTEs),^19^ including those that target EGFR.^20^ To address this limitation, Choi et al.^21^ developed CART.BiTE cells for the treatment of glioblastoma. These engineered T cells co-express an EGFRvIII-specific CAR with an EGFR-specific BiTE, thus combining high tumor specificity (conferred by the EGFRvIII-CAR) with complete tumor eradication (mediated by the EGFR-BiTE locally secreted in the tumor).^21^

**Figure 1:**
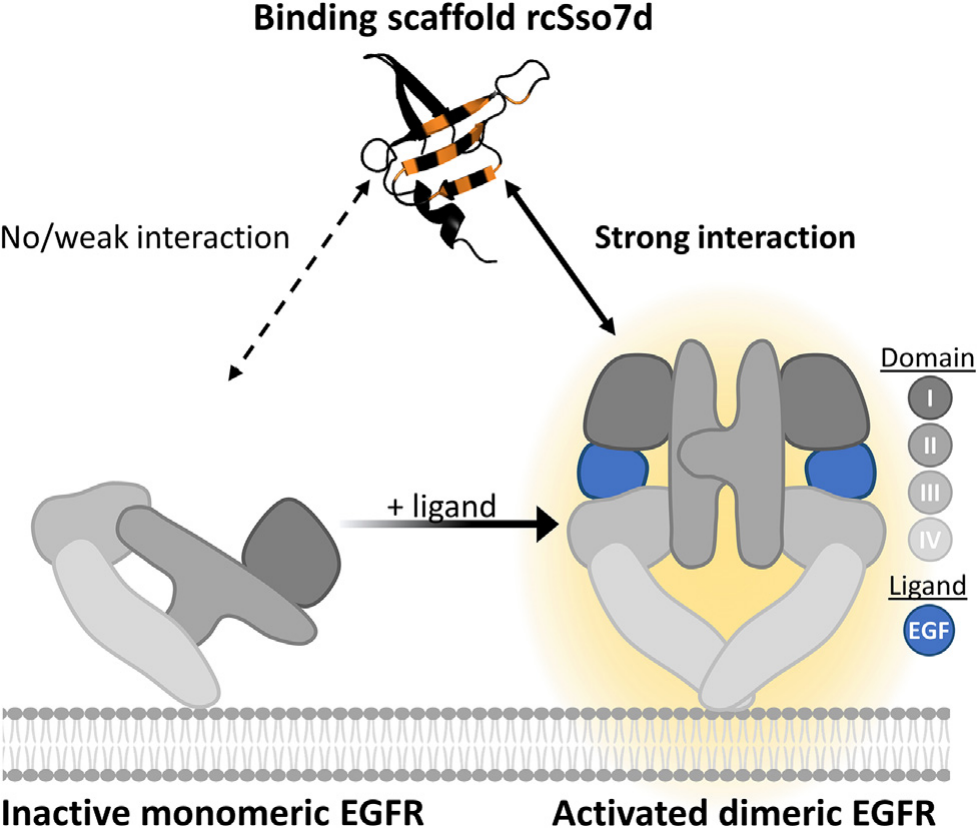
Schematic representation of an engineered binder interacting with activated EGFR Schematic overview of the interaction between the engineered binding scaffold rcSso7d (engineered binding surface in orange; PDB: 1SSO^29^) with inactive monomeric vs. ligand-activated dimeric EGFR. Different gray colors in the schematic EGFR structures represent the four domains of the extracellular part of EGFR. Dimeric EGFR is bound to EGF, shown in blue. Created with BioRender.com.

Nevertheless, to increase the potency of EGFR-targeting therapies such as CAR T cells and BiTEs without further enhancing on-target/off-tumor toxicity, strategies to improve the specificity for EGFR in the tumor tissue are urgently needed. In healthy tissues, EGFR signaling is regulated by tightly controlled secretion of activating EGFR-ligands, which induce a major conformational change in the extracellular domain of EGFR, resulting in EGFR dimerization and activation (Figures 1 and S1A). In contrast, tumor cells have evolved mechanisms to constitutively activate EGFR signaling by (1) EGFR overexpression (leading to ligand-independent activation), (2) acquisition of constitutively activating EGFR mutations, and/or (3) secretion of EGFR ligands,^2^^,^^22^^,^^23^^,^^24^^,^^25^^,^^26^^,^^27^^,^^28^ which results in basal EGFR activation and, as a consequence, a range of cellular outcomes, such as proliferation and resistance to apoptosis.^3^

Therefore, in this study, we sought to develop an engineering strategy to generate CAR antigen binding domains that specifically recognize the activated state of EGFR (Figure 1). In contrast to conventional protein engineering campaigns that have been applied for EGFR targeting,^30^^,^^31^^,^^32^^,^^33^^,^^34^^,^^35^^,^^36^ we specifically directed the selection pressure toward recognition of the ligand-bound, activated conformation of EGFR. Briefly, in consecutive selection rounds, we screened for binding to EGFR in the presence of its ligands and for non-binding to EGFR in the absence of ligands. We successfully engineered several EGFR-binding domains that show pronounced dependency on the presence of EGFR ligands, thus demonstrating their specificity for the activated state of EGFR. Moreover, when integrated into CAR molecules, these binding domains enabled CAR T cells to distinguish between the activated (i.e., ligand-bound) state of EGFR and its inactive conformation.

## Results

### Engineering binding domains to specifically recognize activated EGFR

**Figure 2:**
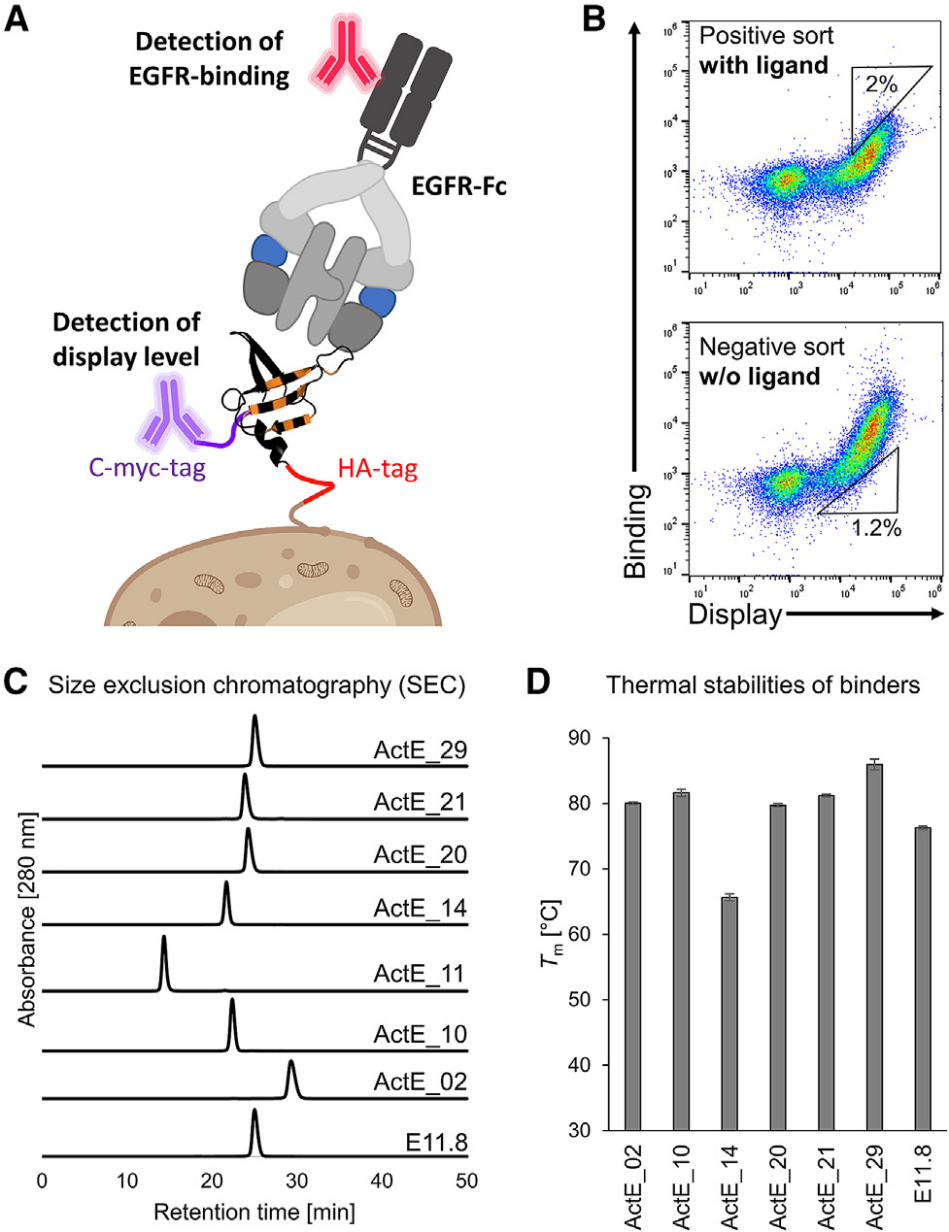
Engineering strategy and biophysical analysis of generated binders (A) Schematic of soluble EGFR-Fc (loaded with EGF, shown in blue) bound to engineered rcSso7d displayed on yeast with monoclonal antibodies for the detection of binding and display level. (B) Representative dot plots with the gating strategy in a positive selection (in the presence of both EGFR-Fc and an EGFR ligand) and a negative selection (presence of EGFR-Fc but absence of EGFR-ligands). (C) SEC profiles of selected ActE-binders and E11.8. One representative of three independent experiments is shown. (D) *T*_m_ values of ActE-binders and E11.8 determined by DSC (mean ± SD of three independent experiments). Created with BioRender.com.

To generate binders that specifically interact with activated, ligand-bound EGFR, we used yeast surface display libraries based on the highly stable protein reduced charge Sso7d (rcSso7d).^36^^,^^37^^,^^38^ In these rcSso7d variants, a rigid β sheet surface is randomly mutated to generate the novel antigen binding site (Figure 1, orange positions in rcSso7d). Briefly, to engineer protein domains with desired binding properties, highly diverse yeast display libraries are typically selected using antigen-coated magnetic beads as well as flow sorting via fluorescently labeled antigen.^36^^,^^39^^,^^40^ In this study, we used the extracellular domain of human EGFR fused to human immunoglobulin G1 (IgG1)-Fc (EGFR-Fc) as soluble antigen for yeast display selection (Figure 2A). Importantly, to direct the selection pressure toward specific recognition of the ligand-bound EGFR-Fc conformation, we developed a screening strategy where we alternated between positive selection (i.e., binding to EGFR-Fc in the presence of ligands) and negative selection (i.e., non-binding to EGFR-Fc in the absence of ligands) (Figures 2B and S1B). In the first positive selection rounds, we used only the ligand EGF, whereas in later rounds, we included a parallel selection arm in which the EGFR-ligand transforming growth factor α (TGF-α) was used instead (Figure S1B). The goal of this alternative selection strategy was the enrichment of binders that interact with the activated conformation of EGFR-Fc irrespective of the type of bound ligand.

After 10 rounds of selection, enriched rcSso7d mutants were sequenced and analyzed individually in the yeast surface display format. Since these binders were engineered to specifically recognize activated EGFR, they were termed ActE_01–ActE_33. Indeed, several of these enriched binders showed enhanced binding to EGFR-Fc in the presence of its ligands EGF or TGF-α (Figures S2A and S3A). Based on their ligand-dependent EGFR binding characteristics and their expression levels (Figures S2 and S3), we chose the seven most promising binders for further analysis (sequences are shown in Figure S4A). In addition, we also tested five previously engineered EGFR binders for ligand dependency. Even though these former selections were conducted in the absence of ligand,^36^ we found one rcSso7d-variant, termed E11.8, that, by coincidence, showed enhanced EGFR binding in the presence of EGF (Figure S4B). Therefore, variant E11.8 was included in this study as well.

### Engineered binders are stable and show ligand-dependent EGFR recognition

To characterize these seven top candidates and E11.8 in detail, they were expressed solubly in *E. coli*. First, they were analyzed with respect to their aggregation properties using high-performance liquid chromatography (HPLC) equipped with a size exclusion chromatography (SEC) column. Most engineered binders were monomeric, with aggregates being virtually undetectable (Figure 2C). The only exception was ActE_11, which showed considerably earlier elution, presumably due to aggregation, and therefore this variant was excluded from further analysis. Second, differential scanning calorimetry (DSC) analysis demonstrated that, despite some destabilization compared with their parental scaffold rcSso7d (melting temperature [*T*_m__]_ of 96°C^36^), which is typical for protein engineering,^41^ – all remaining binders were still highly stable, with *T*_m_ values between 65°C and 86°C (Figure 2D).

**Figure 3:**
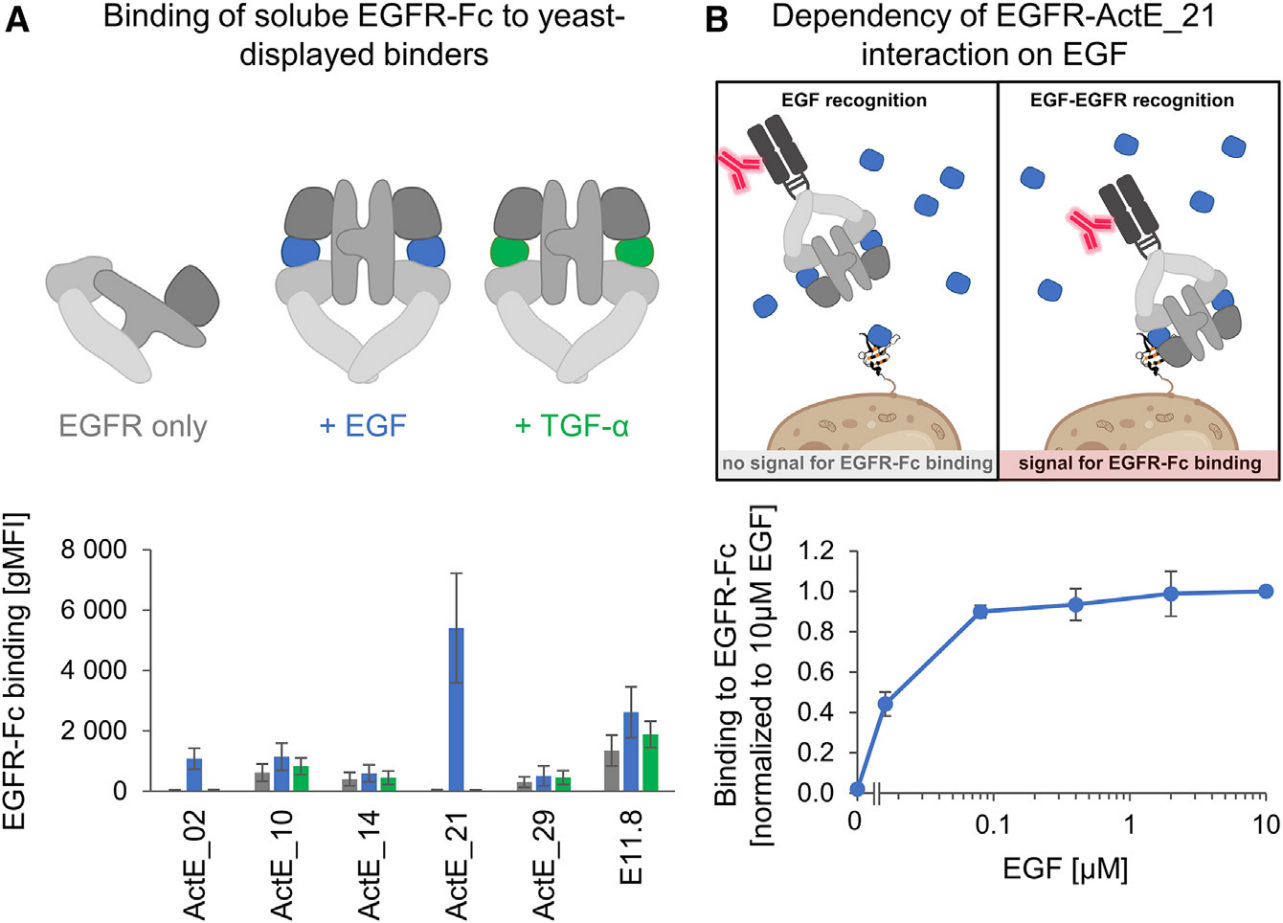
Engineered binders interact with EGFR-Fc in a ligand-dependent manner (A) Binders were displayed on the surface of yeast and tested for binding to 15 nM soluble EGFR-Fc in the absence of EGFR ligands or presence of 100 nM EGF or TGF-α. Binding to EGFR-Fc was measured by flow cytometry, followed by calculation of the geometric mean fluorescence intensity (gMFI). EGFR without EGFR ligands is shown in gray, EGF-loaded EGFR in blue, and TGF-α-loaded EGFR in green (mean ± SD of three independent experiments). (B) Binding of ActE_21 to labeled EGFR as a function of EGF concentration. Binding of ActE_21 to 10 nM soluble labeled EGFR-Fc in the presence of increasing concentrations (0–10,000 nM) of unlabeled EGF. The binding signal is normalized to the highest EGF concentration (mean ± SD of three independent experiments). All gMFI values were background subtracted. Created with BioRender.com.

Next, these monomeric and stable binders were further analyzed with respect to their ligand-dependent EGFR binding properties. Briefly, binders were displayed on the surface of yeast (Figure S4C) and tested for binding to EGFR-Fc in the absence or presence of EGFR ligands (EGF or TGF-α) (Figure 3A). Interestingly, the binders can be classified into two different groups with respect to their ligand dependencies. (1) ActE_02 and ActE_21 showed virtually no binding to ligand-free EGFR (“EGFR only”) or to EGFR in the presence of TGF-α, but EGFR binding was strongly induced by addition of the ligand EGF. (2) In contrast, the other binding domains showed some background binding to EGFR only, which was slightly enhanced by addition of either EGF or TGF-α, indicating that they recognize the active, ligand-bound state of EGFR irrespective of the type of bound ligand (Figure 3A).

For the binders that exclusively bound to EGFR in the presence of the ligand EGF (ActE_02 and ActE_21), there remained the possibility that they only interacted with the ligand without any contribution from the receptor to the binding epitope. To test this possibility, we displayed ActE_21 on the yeast surface and tested for binding to EGFR-Fc. In addition, we added increasing concentrations of EGF, which would be expected to block binding to the receptor at high concentrations if the binder ActE_21 solely interacted with the ligand EGF (Figure 3B). In the absence of EGF, EGFR binding was not detectable, thus confirming the observations in Figure 3A. Importantly, even with a 1,000-fold excess of EGF compared with EGFR-Fc, binding was not reduced (Figure 3B), strongly suggesting that the binder ActE_21 interacts at least partially with EGFR and not only with EGF.

**Figure 4:**
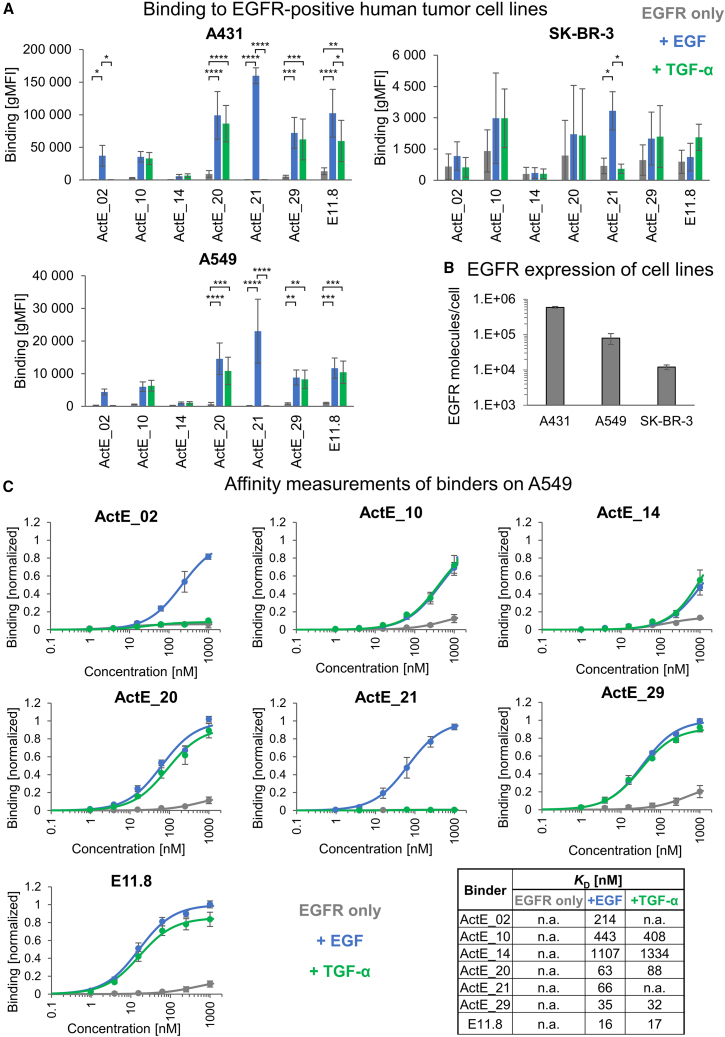
Recognition of EGFR-positive human tumor cells in a ligand-dependent manner (A) EGFR-positive human tumor cell lines were incubated with engineered binders (100 nM, expressed as SUMO fusion proteins) in the absence (gray) or presence of 100 nM EGF (blue) or TGF-α (green), followed by flow cytometry analysis of bound binders (mean ± SD of three independent experiments). Statistical significance was calculated via two-way ANOVA and Sidak’s multiple-comparisons test (∗∗∗∗p < 0.0001, ∗∗∗p < 0.001, ∗∗p < 0.01, ∗p < 0.05). (B) EGFR levels on the surface of A431, A549, and SK-BR-3, measured by flow cytometry and quantification beads (mean ± SD of three independent experiments). (C) Binders were titrated on A549 cells in the absence (gray) or presence of 100 nM EGF (blue) or TGF-α (green). Subsequently, binding intensity was analyzed by flow cytometry. Data were normalized to the maximum signal in the presence of EGF and fitted to a 1:1 binding model to calculate the *K*_D_ values as shown in the table (mean ± SD of three independent experiments). In (A) and (C), all gMFI values were background subtracted. n.a., not analyzable.

Next, we tested the engineered binding domains for recognition of EGFR in its native environment; i.e., the plasma membrane of human cells. We used three human tumor cell lines (A431, A549, and SK-BR-3) expressing different EGFR levels on their surface, ranging from ∼10^4^ – 5 × 10^5^ receptors per cell (Figure 4B). Remarkably, the group of binding domains recognizing ligand-activated EGFR irrespective of the type of bound ligand (ActE_10, ActE_14, ActE_20, ActE_29, and E11.8) showed improved specificity, as demonstrated by the pronounced differences in binding in the presence vs. absence of ligands (Figure 4A). For the other group (ActE_02 and ActE_21), the results closely mirrored those obtained in the yeast display format; i.e., strong dependency of EGFR binding on the presence of the ligand EGF. Moreover, none of our engineered binders showed a detectable binding signal with EGFR-negative human cell lines (Raji and Jurkat), further supporting their pronounced specificity (Figures S5A and S5B). Finally, hardly any cell binding could be observed when using healthy primary human dermal fibroblasts (HDFs) in the absence of ligands and only low-level binding upon addition of EGF (Figures S5A and S5B).

In agreement with the two different binding modes described above (induction by [1] EGF or TGF-α or [2] by EGF only), cross-competition experiments suggest that a representative set of the binders recognizing EGFR upon addition of either EGF or TGF-α binds to a similar or overlapping epitope (ActE_20, ActE_29, and E11.8), whereas ActE_21, which is strictly dependent on EGF, interacts with a different site on EGFR (Figure S5C).

Furthermore, to determine their affinities, the binders were titrated on A549 cells in the presence and absence of ligands. Three binders showed relatively low affinities with *K*_D_ values above 200 nM, while the others bound with higher affinities in the double-digit nM range in the presence of ligand (Figure 4C). Moreover, the pronounced ligand dependencies shown in Figure 4A were again confirmed in these titration experiments.

Together, these data presented above clearly demonstrate that these engineered binding domains interact with EGFR in a ligand-dependent manner—either triggered by different types of ligands (EGF or TGF-α) or solely induced by the presence of EGF. Thus, our engineering strategy indeed enables the generation of binders specifically sensing the activated state of EGFR.

### Generation of a sensitive and specific Jurkat Nur77 reporter cell line

**Figure 5:**
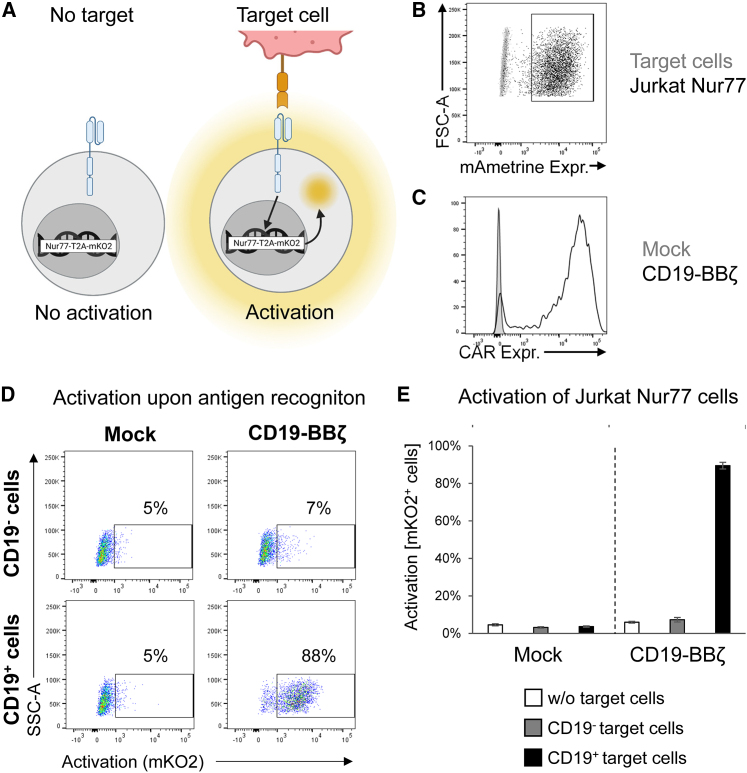
Generation of a Jurkat Nur77 reporter cell line (A) Schematic of the mechanism of the Nur77 reporter cell line. (B) The constitutive expression of mAmetrine by Nur77 reporter cells enables differentiation between target and reporter cells. (C) Flow cytometric analysis of CD19-BBζ CAR expression in Nur77 reporter cells. (D) Representative dot plots depicting the activation (mKO2 expression) of Nur77 reporter cells expressing the CD19-BBζ CAR compared with Mock cells (Nur77 reporter cells not expressing any CAR) in co-culture with CD19^−^ or CD19^+^ target cells. (E) Percentage of activated Nur77 reporter cells expressing the CD19-BBζ CAR compared with Mock cells (no CAR) either alone (without target cells) or after co-culture with CD19^−^ and CD19^+^ target cells (mean ± SD of three independent experiments). Created with BioRender.com. See also Figure S6A.

To test the activation state of CARs in a defined and reproducible manner, we used in *trans* paired nicking^42^ to genetically engineer the human Jurkat T cell line to express a T2A-monomeric Kusabira-Orange 2 (mKO2) reporter cassette downstream of Nur77 (also known as NR4A1), which is an early response gene activated by T cell receptor (TCR) signaling (Figure 5A).^43^^,^^44^^,^^45^^,^^46^^,^^47^ This Jurkat Nur77 reporter cell line was additionally modified to stably express the fluorescent marker monomeric Ametrine (mAmetrine) (Figure 5B). Thus, mKO2 directly linked to Nur77 expression indicates CAR or TCR signaling, whereas mAmetrine is a convenient marker for flow cytometric identification of the Jurkat Nur77 reporter cells in a co-culture with target cells.

To validate the functionality of these Jurkat Nur77 reporter cells, we expressed a standard CD19-specific 4-1BB-based second-generation CAR (CD19-BBζ CAR) (Figure 5C) in these reporter cells. Co-culture experiments with CD19^+^ or CD19^−^ target cells clearly showed antigen-dependent reporter activity (Figure 5D). Data can be quantified with respect to either the percentage of mKO2-positive cells (Figure 5E) or the geometric mean fluorescence intensity (gMFI) of mKO2 (Figure S6A), with both yielding similar results. Taken together, these experiments validate Jurkat Nur77 reporter cells as a clean and reproducible system to quantify CAR signaling.

### Engineered binders enable CAR T cells to specifically recognize ligand-activated EGFR

**Figure 6:**
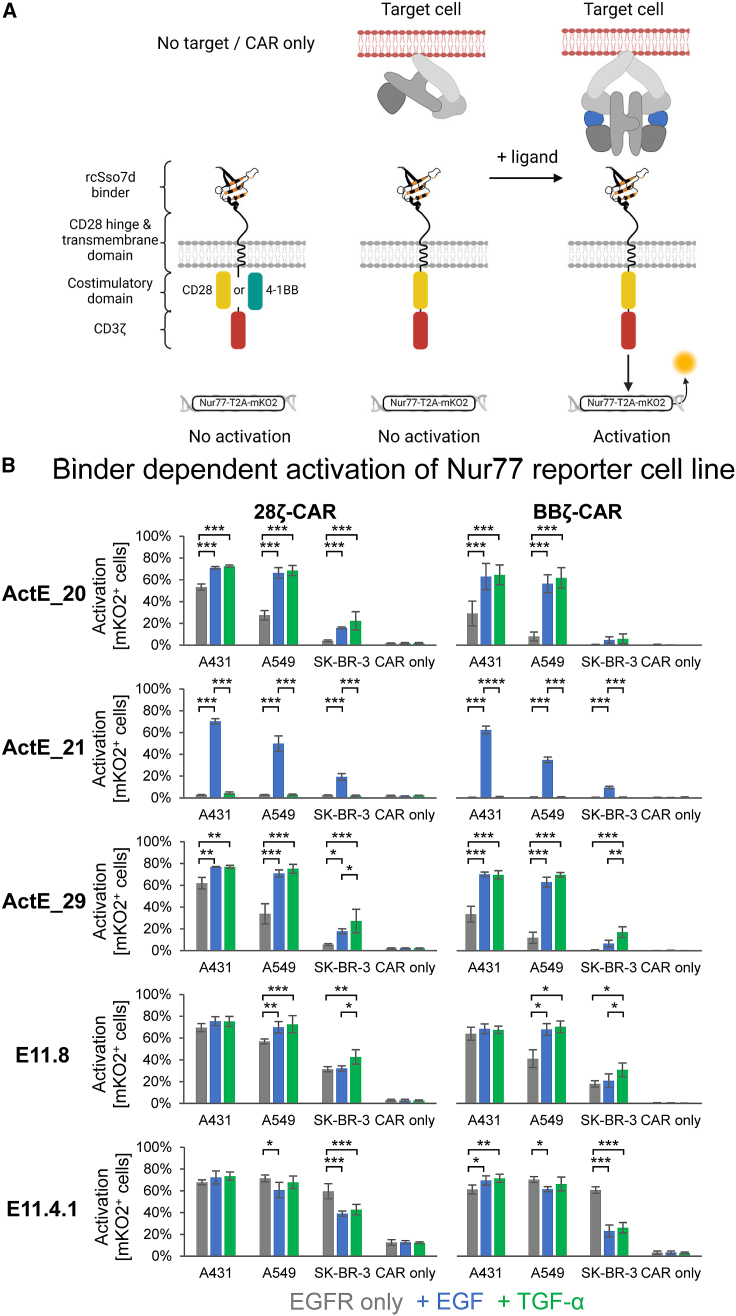
Testing of CARs based on ActE-binders in Jurkat Nur77 reporter cells (A) Schematic of the different components of the CARs and experimental readout. (B) Jurkat Nur77 reporter cells expressing the indicated CARs based on different binders and backbones (BBζ and 28ζ, respectively) alone (CAR only) or in co-culture with target cells in the absence (gray) or presence of 100 nM EGF (blue) or TGF-α (green). The activation level was determined by measuring the mKO2 expression by flow cytometry. Data are presented as mean ± SD of three independent experiments, and statistical significance was calculated via two-way ANOVA with a Tukey post hoc test (∗∗∗p < 0.001, ∗∗p < 0.01, ∗p < 0.05). Created with BioRender.com. See also Figure S6B.

Next, we investigated whether our engineered binders also enable CAR T cells to respond to activated, ligand-bound EGFR. For that purpose, the four most promising binders (ActE_20, ActE_21, ActE_29, and E11.8), which were monomeric and stable and interacted with ligand-activated EGFR with high affinity, were incorporated into second-generation CARs based on CD28 or 4-1BB costimulatory domains (28ζ and BBζ, respectively; Figure S6D). First, we tested these CARs in Jurkat Nur77 reporter cells (Figures 6A and S6B) and additionally included a control binding domain (E11.4.1), which is based on the same scaffold (rcSso7d), but interacts with EGFR in a ligand-independent manner.^36^^,^^48^ While the activation level of this E11.4.1-based control CAR was not elevated upon addition of EGFR ligands, CARs based on ActE_20 and ActE_29 showed significantly improved activity in the presence of EGF or TGF-α (Figure 6B). Even though the ligand-dependent effects were also evident with E11.8-based CAR T cells, they were less pronounced. Remarkably, both ActE_21-based CARs (28ζ and BBζ) were exclusively activated in the presence of EGF, confirming the high specificity of ActE_21 for EGF-bound EGFR.

Taken together, all ActE-binder-based CAR T cells showed ligand-dependent recognition of all three target cell lines, but the achieved activation level correlated with the EGFR surface levels (A431 > A549 > SK-BR-3; Figures 4B and 6B). Thus, these data are in line with the binding analyses described above and clearly demonstrate that these binding domains enable CARs to specifically respond to ligand-activated EGFR.

**Figure 7:**
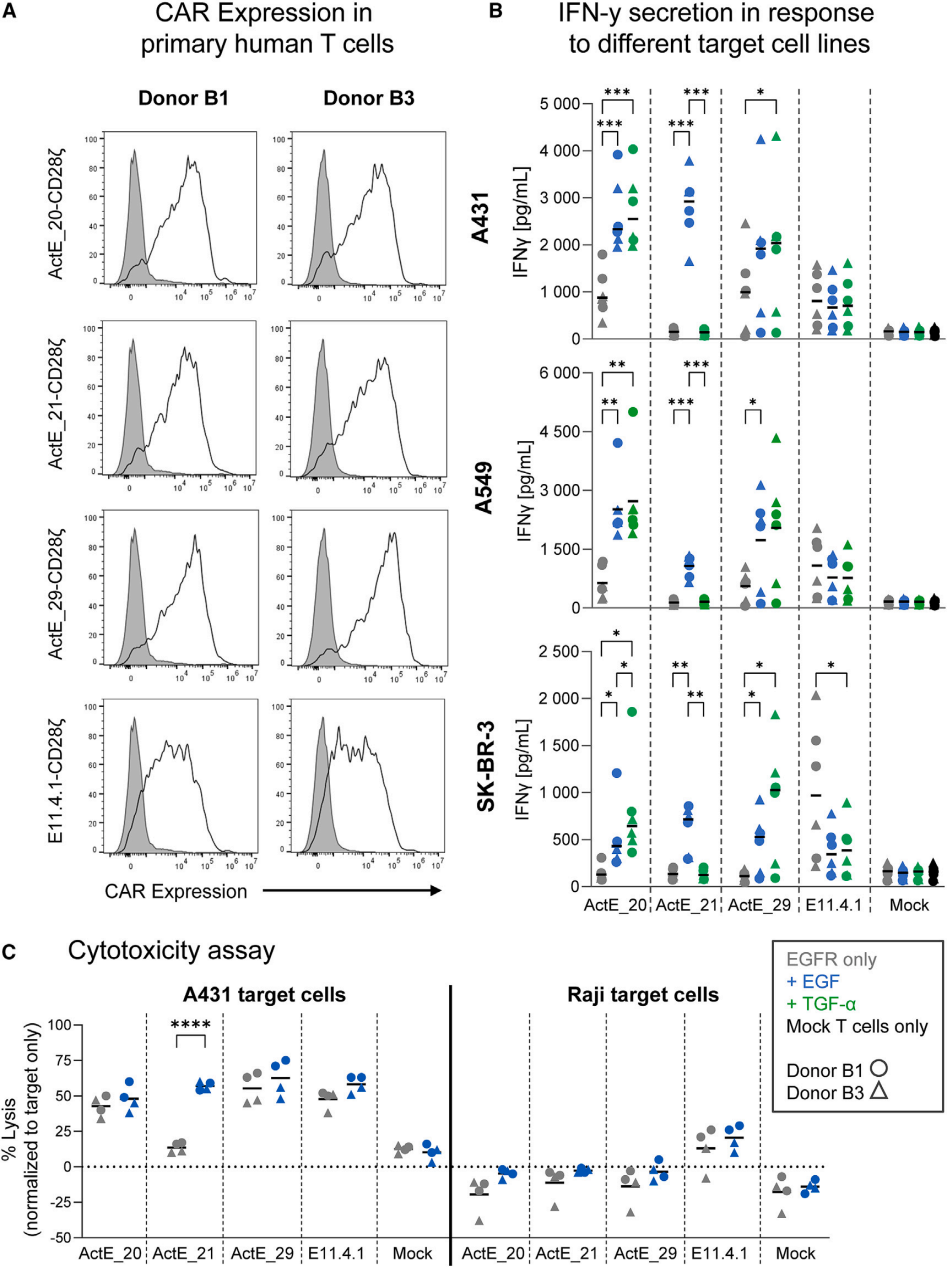
ActE-based CAR T cells specifically respond to ligand-activated EGFR (A) Flow cytometry analysis of CAR expression in T cells from two donors for the indicated CAR constructs. (B) The indicated CAR T cells were co-cultured with target cells at a 1:1 ratio in the absence (gray) or presence of 100 nM EGF (blue) or TGF-α (green) for 4 h, and secreted IFN-γ was measured in the supernatant by ELISA. Mock T cells only (no CAR, black) were not co-cultured with target cells. (C) CAR T cells were co-cultured with target cells at a 2.5:1 effector-to-target (E:T) ratio in the absence (gray) or presence of 100 nM EGF (blue) for 4 h, and lysis was determined by using a luciferase-based assay. Circles and triangles indicate the different donors, B1 and B3, respectively. Statistical significance was calculated via two-way repeated-measures ANOVA with a Tukey post hoc test in (B) (n = 6) and Sidak’s multiple-comparisons test in (C) (n = 4) (∗∗∗∗p < 0.0001, ∗∗∗p < 0.001, ∗∗p < 0.01, ∗p < 0.05). See also Figure S6C.

To further confirm these effects, we performed similar assays with the 28ζ CARs in primary human T cells (Figure 7A). Despite some variability in these assays due to donor-specific variations and slightly different pre-cultivation periods of the T cells, ActE_20 and ActE_29-based CARs again triggered much stronger interferon γ (IFN-γ) secretion in the presence of EGF or TGF-α than in the absence of ligand (Figure 7B). Moreover, the ActE_21-based CAR conferred strictly EGF-dependent release of IFN-γ with all three target cell lines (Figure 7B). Finally, we also assessed CAR T cell activity in cytotoxicity assays (Figure 7C). As expected, EGFR-negative Raji target cells were not lysed by CAR T cells containing ActE binders. When tested with EGFR-expressing A431 targets, ActE_20- and ActE_29-based CAR T cells responded to the EGFR antigen, but ligand-dependency was hardly seen. In contrast, killing by ActE_21-based CAR Ts was only observed upon addition of EGF (Figure 7C), confirming the pronounced dependency of this engineered binding domain on the presence of both EGFR and its ligand EGF.

Summing up, these data demonstrate (1) that the trends observed with Jurkat Nur77 reporter cells were highly similar to those obtained with primary human T cells, thus further validating this Jurkat Nur77 reporter cell line as a rapid, sensitive, and reproducible tool to analyze CAR signaling, and (ii) that our engineered binders enable CAR T cells to specifically recognize the activated state of EGFR.

## Discussion

In this study, we developed a protein engineering strategy enabling the generation of binding domains that specifically interact with the ligand-activated state of EGFR. We observed this reproducible ligand-dependent EGFR interaction in several different experimental systems: (1) binding of yeast displayed binders to soluble EGFR-Fc, (2) soluble binders interacting with EGFR-positive human cancer cell lines, (3) response of Jurkat Nur77 reporter CAR T cells and (4) of primary human CAR T cells to EGFR-positive human target cells.

As an initial proof of concept, we chose the rcSso7d scaffold platform, which provides several critical advantages: (1) the original protein rcSso7d is small (7 kDa) and highly stable with a *T*_m_ of 96°C;^36^ (2) it contains a small, flat, and rigid binding surface (Figure 1); (3) most binders derived from rcSso7d are resistant to aggregation;^36^^,^^49^^,^^29^ and (4) these engineered binding domains have been shown to be well expressed in different hosts, including human T cells.^48^^,^^29^ In line with these previous observations, we also observed high stability and low aggregation tendencies of our engineered binders as well as efficient expression in human T cells when being fused to CAR backbones. A potential disadvantage of rcSso7d is its non-human origin (*Sulfolobus solfataricus*), thus raising the risk of immunogenicity when being used in therapeutics. However, we note that rcSso7d is very small with only 61 amino acids, which is only slightly above the typical number of non-human amino acid positions found in humanized single-chain variable fragments (scFvs).^50^

Given the broad distribution of EGFR expression in many healthy human tissues, EGFR-targeting strategies with improved tumor specificity are required to be able to improve CAR T cell potency without simultaneously enhancing on-target/off-tumor toxicity. One potential approach is the specific targeting of the tumor-specific deletion variant EGFRvIII. Jungbluth et al.^33^ generated an EGFRvIII-specific murine mAb by immunizing mice with a cell line expressing human EGFRvIII. By coincidence, one of the obtained mAbs (mAb806) also showed preferential binding to human cells overexpressing wild-type EGFR.^33^ It was found that mAb806 preferentially binds EGFR upon overexpression or in the EGFRvIII deletion mutant, but it interacts neither with the tethered, inactive state nor with the fully ligand-bound, dimeric conformation of EGFR.^33^^,^^51^^,^^52^ Based on the promising observations that overexpressed EGFR and EGFRvIII, both of which being tumor associated, are preferentially recognized, Ravanpay et al.^14^ incorporated an 806-based scFv into a CAR backbone and demonstrated that these EGFR806-CAR T cells conferred high anti-tumor potency in a glioblastoma *in vivo* model but no on-target/off-tumor activation in EGFR-positive human teratomas in the same animals.^14^ Recently, a phase I clinical study with EGFR806-CAR T cells showed mixed responses in three of 11 patients with recurrent/refractory solid tumors, with only one patient experiencing severe toxicities.^9^ Thus, these studies on mAb806 collectively highlight the benefit of binding entities that recognize overexpressed and/or activated states of EGFR.

While our engineered binding domains also recognize an activated conformation of EGFR, they show different binding modes. In contrast to mAb806, they interact with the ligand-bound state of EGFR. Interestingly, even though all of our binders preferentially bound to EGFR in the presence of EGFR ligands, our engineering strategy yielded two types of binding mechanisms: (1) recognition of the activated conformation of EGFR independent of the type of bound ligand (EGF or TGF-α) or (2) interaction with EGFR only in the presence of a particular ligand. Of note, the binders specifically interacting with EGFR only in the presence of EGF (ActE_02 and ActE_21) showed virtually absent background binding to EGFR in the absence of ligands as well as in the presence of another ligand (TGF-α). This extremely high specificity could have been explained by exclusive interaction with the ligand EGF. However, we also demonstrated that even a 1,000-fold excess of free EGF did not block binding of ActE_21 to EGFR. Thus, despite its high dependency on the presence of EGF, this binder does not solely interact with EGF.

Similarly, the binders recognizing the activated conformation (e.g., ActE_20 and ActE_29) showed hardly any background binding in the absence of ligands (Figures 4A and 4C). However, this background activity was enhanced in the CAR assays (Figures 6 and 7), presumably due to the high antigen sensitivity of CAR T cells. Therefore, we conclude that the best specificity is obtained with binders interacting with a certain ligand-EGFR complex, such as ActE_21, which recognizes EGF-EGFR.

A potential limitation of targeting ligand-activated EGFR could be the well-known ligand-induced internalization of EGFR, which, of course, results in lower surface levels that can be recognized by the CAR T cell. Of note, while EGF induces rapid and strong receptor downregulation, this effect is less pronounced with other ligands, such as TGF-α,^53^^,^^54^^,^^55^^,^^56^ which was also observed in our experiments (Figure S7A). Moreover, we observed efficient CAR T cell activation, demonstrating that sufficient amounts of ligand-bound EGFR remain on the cell surface. Thus, ligand-induced receptor downregulation reduces the number of available antigens on the cell surface to a certain extent, but this effect does not preclude CAR T cell recognition.

Another potential concern could be binder-induced EGFR activation, since our engineered binding domains bind to (and therefore possibly stabilize) the active conformation of EGFR. However, when tested in EGFR signaling assays, none of the binders triggered or enhanced EGFR signaling, neither in the absence nor in the presence of the ligand EGF (Figure S7B). Thus, even though these binders specifically interact with active EGFR, we did not observe any binder-mediated EGFR phosphorylation.

In this study, we introduce a protein engineering concept for the generation of binding domains specifically recognizing the ligand-activated state of EGFR. We confirm that the obtained binding domains show pronounced ligand dependency and conformation specificity in several different experimental systems. Moreover, we demonstrated that, when being incorporated into CARs, these binding domains enable CAR T cells to specifically recognize the activated state of EGFR on target cells. Since many human receptors undergo conformational changes upon ligand activation,^57^^,^^58^ we anticipate that this engineering concept will be broadly applicable for the generation of binders and CAR T cells directed against activated (i.e., tumor-associated) receptor states, thus representing a major step forward to improve the tumor specificity of CAR T cell therapies.

### Limitation of the study

The engineering strategy introduced in this study allows for efficient generation of binding domains recognizing the activated state of EGFR. However, for receptors that do not (or only slightly) change their conformation, it may be more difficult to engineer binders that differentiate between the ligand-activated and the resting state of the respective receptor. Moreover, many receptors are difficult to express in their native conformation. As a consequence, the recombinant extracellular domains of those receptors are often partially misfolded, resulting in enrichment of binders that might not interact with the natural, membrane-embedded receptor, which is a well-known limitation in the protein engineering field.^35^^,^^59^ To overcome this potential limitation, our approach could also be adapted to cell panning selections using mammalian cells as targets. Nevertheless, our study clearly demonstrates that this presented engineering strategy is very effective when using well-folded, high-quality receptor antigens.

## STAR★Methods

### Key resources table

**Table:** 

REAGENT or RESOURCE	SOURCE	IDENTIFIER
**Antibodies**		

Anti-human EGFR PE (clone AY13)	BioLegend	Cat# 386303 (also 386304), RRID:AB_2941568
Penta-His Alexa Fluor 647 conjugate	Qiagen	Cat# 35370, RRID:AB_3083468
Penta-His Alexa Fluor 488 conjugate	Qiagen	Cat# 35310, RRID:AB_3083465)
Mouse anti-*c*-myc (clone 9E10)	Thermo Fisher Scientific	Cat# 13–2500, RRID:AB_2533008
Goat anti-Mouse IgG Alexa Fluor 488	Thermo Fisher Scientific	Cat# A-11001,RRID:AB_2534069
Anti-podoplanin-PE (clone PMab-1)	Novus Biologicals	Cat# NBP3-11971PE, RRID:AB_3083466
anti-DYKDDDDK Tag APC (clone L5)	BioLegend	Cat# 637308 (also 637307), RRID:AB_2561497
Anti-CD3 VioGreen™ (clone REA613)	Miltenyi Biotec	Cat# 130-113-142, RRID:AB_2725970
Anti-CD4 PerCP (clone OKT4)	BioLegend	Cat# 317431, RRID:AB_2028492
Anti-CD8 FITC (clone HIT8a)	Immunotools	Cat# 21810083, RRID:AB_3083535
Anti-HA.11-AF488 (clone 16b12)	BioLegend	Cat# 901509, RRID:AB_2565072
Anti-HA.11-AF647 (clone 16b12)	BioLegend	Cat# 682404, RRID:AB_2566616)
Anti-rabbit F(ab’)2 Fragment AF647	Cell Signaling Technology	Cat# 4414S, RRID:AB_3083475
Phospho-EGF Receptor (Tyr1068) antibody (clone D7A5)	Cell Signaling Technology	Cat# 3777, RRID:AB_2096270

**Bacterial and virus strains**		

*E. coli*: Tuner (DE3)	Novagen	Cat# 70623

**Biological samples**		

Buffy coat for isolation of human T cells	Austrian Red Cross	Unknown sex and age
Primary human dermal fibroblasts	Evercyte GmbH	Donor #76, female, 58 years

**Chemicals, peptides, and recombinant proteins**		

Animal-free recombinant human EGF	Peprotech	Cat# AF-100-15, Gene ID: 1950
Animal-free recombinant human TGF-α	Peprotech	Cat# AF-100-16A, Gene ID: 7039
Recombinant human IL-2	Peprotech	Cat# 200-02
dPTP	Jena Bioscience	Cat# NU-1119S
8-oxo-dGTP	Jena Bioscience	Cat# NU-1117S
IVISbrite D-Luciferin K+ Salt	Perkin Elmer	Cat#122799
Streptavidin-AF647	Thermo Fisher Scientific	Cat# S32357

**Critical commercial assays**		

ELISA MAX Deluxe Set Human IFN-γ	BioLegend	Cat# 430115
NucleoBond Xtra Midi	Macherey-Nagel	Cat# 740410.50
EZ-Link Sulfo-NHS-LC-LC-Biotin	Thermo Fisher Scientific	Cat# 21338
Zymoprep Yeast Plasmid Miniprep II Kit	Zymo Research	Cat# D2004
RosetteSep Human T cell enrichment cocktail	STEMCELL Technologies	Cat# 15021
QuantiBRITE Phycoerythrin (PE) Fluorescence Quantitation Kit	Becton Dickinson	Cat# 340495

**Experimental models: Cell lines**		

A431	ATCC	ATCC CRL-1555
A549	ATCC	ATCC CCL-185
SK-BR-3	ATCC	ATCC HTB-30
A431 GFP/luciferase	Made in-house	N/A
Raji GFP/luciferas	Made in-house	N/A
Raji	Gift from Dr. Sabine Strehl	https://ccri.at/research-group/sabine-strehl-group/
Jurkat E6.1	Gift from Dr. Michael Dworzak	https://ccri.at/research-group/michael-dworzak-group/
Jurkat Nur77 reporter cell line	This paper	N/A
HEK293-6E	NRC Biotechnology Research Institute, Canada	http://www.nrc-cnrc.gc.ca/eng/; RRID VCL_: CHF20

**Experimental models: Organisms/strains**		

*S. cerevisiae:* EBY100	ATCC	ATCC MYA-4941

**Recombinant DNA**		

pE-SUMO	LifeSensors	Cat# PE-1106-0020
pCTCON2V	Genscript	N/A
pTT5	NRC Biotechnology Research Institute, Canada	http://www.nrc-cnrc.gc.ca/eng/
pTT5-EGFR-4xG4S-Fc	In this paper	N/A
pTT5-EGFR-1xG4S-Fc	In this paper	N/A

**Software and algorithms**		

GraphPad Prism v10.1.1	GraphPad Software	RRID: SCR_002798
FlowJo v10.8.1	BD Life Sciences	RRID: SCR_008520
Biorender	Biorender	RRID:SCR_018361

**Other**		

Yeast-display library rcSso7d-11	Traxlmayr et al.^36^	N/A
Yeast-display library rcSso7d-18	Traxlmayr et al.^36^	N/A

### Resource availability

#### Lead contact

Further information and requests for resources and reagents should be directed to and will be fulfilled by the lead contact, Michael W. Traxlmayr (michael.traxlmayr@boku.ac.at).

#### Materials availability

The cell lines and plasmids will be made available by the lead contact upon request.

#### Data and code availability

• All data reported in this paper will be shared by the lead contact upon request.
• This paper does not report original code.
• Any additional information required to reanalyze the data reported in this paper is available from the lead contact upon request.

### Experimental model and study participant details

#### Cell culture

Buffy coats from anonymous healthy donors (unknown sex and age) were commercially obtained from the Austrian Red Cross, Vienna. Primary human dermal fibroblasts (HDFs) from donor #76 (female, 58 years) were obtained from Evercyte GmbH. Primary CD3^+^ T cells were isolated from buffy coats using the RosetteSep Human T cell enrichment cocktail (STEMCELL Technologies) according to the manufacturer's instructions and cryopreserved in RPMI-1640 GlutaMAX medium (Thermo Fisher Scientific) supplemented with 20% FBS superior and 10% DMSO (both from Sigma Aldrich). T cells were activated with Human T-Activator CD3/CD28 Dynabeads (Thermo Fisher Scientific) and cultured in RPMI GlutaMAX supplemented with 10% FBS superior, 1% penicillin-streptomycin solution (10,000 U/mL, Thermo Fisher Scientific) and 200 U/mL IL-2 (Peprotech). Activated T cells were split every second day and cultured at densities between 0.3 and 1 × 10^6^ cells/mL. Raji, Jurkat E6.1 cells (gift from Dr. Sabine Strehl and Dr. Michael Dworzak, respectively, CCRI Vienna), Raji GFP/luciferase (made in-house) and Jurkat Nur77 reporter cells (generated in this study) were cultured in the same medium as primary T cells but without additional IL-2 and split every other day. A431 GFP/luciferase (made in-house), A431, A549 and SK-BR-3 (all from ATCC) were cultured in RPMI-1640 GlutaMAX supplemented with 10% FBS superior and 1% penicillin-streptomycin solution and passaged every 2–4 days. Primary HDFs were cultured in Dulbecco′s Modified Eagle′s Medium/Nutrient Mixture F-12 Ham (Sigma Aldrich) supplemented with 10% FBS superior and 4 mM L-glutamine (Thermo Fisher Scientific). All cells were cultured at 37°C, 5% CO_2_ and 97% humidity. HEK293-6E cells (NRC Biotechnology Research Institute, Canada) were cultivated in FreeStyle F17 expression medium containing 0.1% Pluronic F-68, 4 mM L-glutamine (all from Thermo Fisher Scientific) and 25 μg/mL G418 (Merck KGaA) in a Climo-Shaker ISF1-XC (Adolf Kühner AG) at 130 rpm and 37°C, 7% CO_2_ and 80% humidity.

### Method details

#### Expression and purification of soluble EGFR-Fc

The extracellular part of human EGFR (Uniprot P00533, aa 1–645) was fused to human IgG1-Fc (Uniprot P01857, aa 103–330) which was cloned into a pTT5 vector (NRC Biotechnology Research Institute, Canada). Two constructs were made with different linker lengths (1xG_4_S and 4xG_4_S, respectively) between EGFR and IgG1-Fc to ensure enough flexibility for EGFR dimerization (pTT5-EGFR-1xG_4_S-Fc and pTT5-EGFR-4xG_4_S-Fc). Additionally, both constructs were equipped with a biotin acceptor peptide^60^ and a hexahistidine (His_6_)-tag. For the transfection, pTT5 vectors (pTT5-EGFR-1xG_4_S-Fc and pTT5-EGFR-4xG_4_S-Fc, respectively) were prepared using the NucleoBond Xtra Midi kit (Macherey Nagel) in accordance with the manufacturer’s instructions. HEK293-6E cells were transiently transfected with 1 μg/mL plasmid DNA and 2.5 μg/mL PEI MAX solution (Polysciences Europe GmbH) at a cell density of approximately 1.7 × 10^6^ cells/mL. After 48 h, the cells were fed with 0.5% (w/v) tryptone N1 (Organotechnie) and 0.25% (w/v) glucose. Culture supernatants were harvested by two centrifugation steps (500 g, 10 min, 4°C and 17,000 g, 20 min, 4°C, respectively) 120 h post-transfection before being filtered (0.45 μm PVDF membrane filter, Merck KgaA) and applied onto a 5 mL HiTrap rProtein An FF column (Cytiva) pre-equilibrated with running buffer (20 mM phosphate buffer with 200 mM NaCl, pH 7.4). The protein was eluted with 0.1 M glycine solution, pH 3.3, and immediately afterward, 2 M TRIS-buffer, pH 12, was added to neutralize the acidic pH. The collected proteins were dialyzed three times against running buffer in a pre-wetted SnakeSkin dialysis tubing (10,000 MWCO, Thermo Fisher Scientific) at 4°C overnight before being concentrated using Amicon Ultra-15 centrifugal filter units (50,000 MWCO, Merck KgaA). Subsequently, a portion was biotinylated using the EZ-Link Sulfo-NHS-LC-LC-Biotin kit (Thermo Fisher Scientific) in accordance with the manufacturer’s instructions, before both the biotinylated and the non-biotinylated samples were applied separately onto a HiLoad 16/600 Superdex 200 pg column (Cytiva) pre-equilibrated with the same running buffer as before. The proteins were stored at −80°C.

#### Screening for binders using yeast display

Based on rcSso7d, two previously established yeast-display libraries (rcSso7d-11 and rcSso7d-18, respectively^36^), with a diversity of 1.4 × 10^9^ each, were displayed on *S. cerevisiae* strain EBY100 (ATCC). The handling of the libraries regarding thawing and dilution in SD-CAA medium, the induction of yeast surface expression in SG-CAA medium as well as the incubation of the induced naive libraries with Dynabeads Biotin Binder (Thermo Fisher Scientific) loaded with biotinylated EGFR-Fc in the presence of 20 nM EGF (Peprotech) was done as described previously (positive selection).^40^^,^^61^ In the second round, cells were incubated three times with bare beads to deplete non-specific binders (negative selection) before another positive selection. After the second enrichment, plasmids were isolated (Zymoprep Yeast Plasmid Miniprep II Kit, Zymo Research) and served as a template for an error prone PCR with 2 μM of the nucleotide analogs dPTP and 8-oxo-dGTP (both from Jena Bioscience). Subsequently, the resulting product was amplified with Q5 high-fidelity DNA polymerase (New England Biolabs, NEB) and used as an insert for the following electroporation of EBY100 cells together with the linearized pCTCON2V vector (Genscript) as described in Chen et al. 2013.^61^ After a third round of bead selection, which included three negative and one positive selection, the subsequent rounds of enrichment were carried out through flow sorting. In positive selections, yeast cells were stained with different concentrations (depending on the selection round) of soluble EGFR-Fc in the presence of 20x molar excess of EGF or TGF-α (Peprotech) in PBSA (PBS supplemented with 0.1% BSA (Sigma-Aldrich)). On the other hand, negative selections were carried out in the presence of EGFR-Fc, but absence of EGFR ligands. 1–3 x 10^7^ induced yeast cells were washed twice with ice-cold PBSA and stained as described above at 4°C for 1 h while shaking. For stainings with biotinylated soluble EGFR-Fc, additionally 0.5 μg/mL mouse anti-*c*-myc antibody (clone 9E10, Thermo Fisher Scientific) was added to detect the surface level of binders on yeast cells. After a washing step, cells were stained with 20 μg/mL streptavidin-Alexa Fluor (AF) 647 and 20 μg/mL anti-mouse IgG-AF488 (both Thermo Fisher Scientific) at 4°C for 20 min while shaking. In contrast, for non-biotinylated soluble EGFR-Fc stainings, the secondary staining was done with 5 μg/mL Penta-His antibody (AF488 or AF647, Qiagen) for detection of EGFR-Fc, and anti-HA.11 epitope tag antibody (AF488 or AF647, clone16B12, Biolegend) at 2.5 μg/mL or 1.25 μg/mL, respectively, for detection of display levels. Subsequently, cells were washed twice with ice-cold PBSA and resuspended in ice-cold PBSA just before the cell sorting either using a FACS Aria Fusion cell sorter (BD Biosciences) or SH800S cell sorter (Sony Biotechnology). All steps were performed on ice or 4°C.

#### Yeast display for binding analysis of single binders

Based on their sequence, 33 enriched binders were selected for transformation of EBY100 cells with the Frozen-EZ Yeast Transformation II kit (Zymo Research) according to the manufacturer’s instructions. Binding of single clones to different concentrations of soluble EGFR-Fc in the presence or absence of EGFR ligands was analyzed as described above, but only 1-3 x 10^6^ cells in 50 μL in V-bottom 96-well plates were used. For the titration of EGF, yeast cells displaying ActE_21 were incubated with 10 nM soluble non-biotinylated EGFR-Fc and increasing concentrations of EGF (0–10,000 nM) at 4°C for 60 min on a shaker. Secondary staining was carried out in 25 μL as described above. The measurement was done on a Cytoflex S instrument (Beckman Coulter). Data were analyzed with FlowJo software (BD Life Sciences).

#### Soluble expression of engineered binding domains

Binders were sub-cloned into the pE-SUMO-vector (LifeSensors) for expression as fusion proteins with His_6_-tagged small ubiquitin-like modifier (SUMO). After transformation of Tuner (DE3) *E. coli* with sequence-verified plasmids, cultures were incubated in LB medium supplemented with 50 μg/mL kanamycin at 37°C. Cells were diluted in terrific broth (12 g/L tryptone, 24 g/L yeast extract, 4% glycerol, 2.31 g/L KH_2_PO_4_ and 16.43 g/L K_2_HPO_4_∗3H_2_O) supplemented with kanamycin to an OD_600_ of 0.1 and further incubated at 37°C. Expression was induced at an OD_600_ of 1.2 by addition of 1 mM isopropyl β-D-1-thiogalactopyranoside (IPTG) at 20°C. After overnight expression, cells were harvested (5,000 g, 20 min, 4°C) and resuspended in sonication buffer (50 mM phosphate buffer, 0.3 M NaCl, 3% glycerol, 1% Triton X-, pH 8). After sonication and centrifugation (20,000 g, 30 min, 4°C), the supernatant was supplemented with 10 mM imidazole and applied twice on TALON metal affinity resin (Takara Clonetch). The resin was washed twice with equilibration buffer (50 mM phosphate buffer, 0.3 M NaCl, pH 8) supplemented first with 10 mM imidazole and then with 15 mM, followed by elution with equilibration buffer supplemented with 250 mM imidazole. After dialysis in PBS, the binders were either directly frozen as SUMO-fusions or digested with SUMO-protease overnight at 22°C, resulting in the cleavage of His_6_-SUMO from the respective binder. To separate the binder from His_6_-SUMO, a preparative SEC was done in PBS using a HiLoad 16/600 Superdex 75 pg column (Cytiva). Collected fractions with the binder were concentrated using Amicon Ultra-15 centrifugal filter units (3,000 MWCO) and stored at −80°C.

#### Differential scanning calorimetry (DSC)

DSC experiments were performed with the MicroCal PEAQ-DSC (Malvern Panalytical). 50 μM of the respective binder in PBS were heated up from 20°C to 110°C with a heating rate of 1°C/min. Data analysis was performed with the MicroCal PEAQ-DSC software (Malvern Panalytical). After buffer baseline subtraction and normalization for protein concentration, transitions were fitted with a non-two-state unfolding model.

#### Analytical size exclusion chromatography (SEC)

Binders were diluted in running buffer (PBS supplemented with 200mM NaCl) to a concentration of 1 mg/mL and filtered through a 0.1 μm Ultrafree MC VV centrifugal filter (Merck KGaA). Subsequently, 100 μg of the respective binders were applied to a Superdex 75 column (10/300, Cytiva) connected to an HPLC Prominence LC20 System (Shimadzu) at a flow rate of 0.75 mL/min at 25°C.

#### Construction of the Jurkat Nur77 reporter cell line

In trans paired nicking^42^ was employed to genetically modify the Nur77 (also known as NR4A1) allele between exon 7 and the stop-codon to insert a T2A-mKO2 cassette. The pMA backbone donor vector (GeneArt, Thermo Fisher Scientific) contained an insert consisting of a T2A ribosomal skipping sequence in frame with the fluorescent protein mKO2, flanked by Nur77 CRISPR target sites and homology arms of 500 bp for the 5’ (5′HA) and 3′ end (3′HA). The co-transfected CRISPR/Cas9 ribonucleoprotein complex (RNP) consisted of Alt-R crRNA, tracrRNA, and Cas9/D10A nickase V3 recombinant protein (all from IDT). Following guide RNA was used for targeting the genomic locus: 5′-CCGTGGACTAAAGGCACATG-3′. 10^6^ Jurkat E6.1 cells were electroporated using an Amaxa Nucleofector 2b (program X-005) and Nucleofection V Kit (Lonza), 5 μg circular donor plasmid and 250 pmol RNP. Following the expansion of surviving cells, edited cells were stimulated (10 ng/mL PMA and 2.5 μM Ionomycin) for 24 h, followed by flow cytometric single-cell sorting based on mKO2 signal. Clones were expanded, tested for mKO2 upregulation upon stimulation and genotyped to confirm heterozygous integration of the T2A-mKO2 cassette.

The Jurkat Nur77 reporter cell line expressing mAmetrine was established by transduction with a third-generation pCDH-based lentiviral vector encoding mAmetrine and LNGFR (CD271) (System Biosciences). Preparation of lentiviral particles was described previously.^48^ Transduced cells were selected using the MACSelect LNGFR System (Miltenyi Biotec), according to manufacturer’s instructions.

#### Design of CARs and transfection of Jurkat Nur77 reporter cells and primary human T cells

The constructs were designed for *in vitro* transcription and electroporation. Therefore, the gene fragments consist of a T7 promotor, a Kozak sequence followed by a signal peptide and the CAR. The CARs themselves consist of the engineered binding domain with a 1xG4S linker before a MAP tag,^62^ followed by a CD28 hinge and transmembrane domain, a CD28 or 4-1BB costimulatory domain and a CD3ζ signaling domain (Figure S6D). The CD19-BBζ CAR construct contains the GM-CSF-Rα signal peptide (Uniprot P15509, aa 1–22) followed by the scFv of the FMC63 antibody, a FLAG tag,^63^ a CD8a hinge and transmembrane domain, a 4-1BB costimulatory domain and a CD3ζ signaling domain (Figure S6D). CAR genes were constructed by Gibson Assembly (NEB) according to the manufacturer's instructions and amplified by PCR with Q5 high-fidelity DNA polymerase. Subsequently, the sequence-verified PCR product served as template for *in vitro* transcription with the HiScribe T7 ARCA mRNA kit (with tailing) followed by purification with the Monarch RNA Cleanup kit (both from NEB) following the manufacturer’s instructions. Before the electroporation, cells were washed first with RPMI-1640 (without phenol red, Thermo Fisher Scientific) and then with Opti-MEM (reduced serum medium without phenol red, Thermo Fisher Scientific) (300 g, 7 min, 20°C). Finally, 6 × 10^6^ Jurkat Nur77 reporter cells or 8 × 10^6^ primary T cells were electroporated with 4 μg mRNA in 100 μL Opti-MEM using the square wave protocol (500 ms, 4 mm cuvettes, 3 ms or 5 ms, respectively) of the Gene Pulser Xcell Electroporation system (Bio-Rad). After electroporation, cells were immediately transferred to pre-warmed culture medium. For the cytotoxicity assays, cells were transferred into RPMI-1640 (without phenol red) supplemented with 10% FBS superior, 1% penicillin-streptomycin solution, and 200 U/mL IL-2.

#### Flow cytometric analysis of CAR expression and T cell phenotype

Cells were counted and then washed with ice-cold PBSA (300 g, 5 min, 4°C). 10^5^ cells were first incubated with 10% human serum (PAN-Biotech) in PBSA for blocking (4°C, 10 min) before addition of anti-podoplanin antibody (detection of MAP-tag, PE, clone PMab-1, Novus Biologicals, 1:100 dilution) or anti-DYKDDDDK antibody (detection of the FLAG tag, APC, clone L5, BioLegend, 1:167) for detection of CAR expression. To determine the phenotype, primary human T cells were stained with the following antibodies: anti-CD3 (VioGreen, clone REA613, Miltenyi, 1:200), anti-CD4 (PerCP, clone OKT4, BioLegend, 1:100) and anti-CD8 (FITC, clone HIT8a, Immunotools, 1:67). All stainings were performed in 50 μL. After staining (4°C, 20 min), cells were washed twice with 200 μL ice-cold PBSA, pelleted (300 g, 5 min, 4°C), and resuspended in 80 μL ice-cold PBSA just before the measurement with a Cytoflex S instrument. Data were analyzed with FlowJo software.

#### Binding to EGFR-positive cells

Cells were either detached with Trypsin/EDTA and counted (A431, A549, SK-BR-3, primary human dermal fibroblasts) or directly counted (Raji, Jurkat). Next, cells were washed with PBSA, centrifuged (300 g, 7 min, 4°C), and resuspended to the desired concentration in ice-cold PBSA. 10^5^ cells in 33 μL PBSA were transferred into a V-bottom 96-well plate and stained with 33 μL binder (300 nM; expressed as SUMO-fusion protein) either with 33 μL PBSA (absence of ligand) or 33 μL of EGF or TGF-α (300 nM), resulting in a 1:3 dilution and a final concentration of 100 nM binder and 100 nM ligand, respectively. Cells were incubated at 4°C for 1 h. To determine the binding affinities of soluble binders on A549, 10^5^ cells in 25 μL were transferred into a V-bottom 96-well plate and stained with 25μL His_6_-SUMO-tagged binders (final concentration 0–1,000 nM) alone or pre-mixed with EGF or TGF-α (final concentration 100 nM), respectively, at 4°C for 1 h. Subsequently, cells were washed with 200 μL ice-cold PBSA, centrifuged (300 g, 7 min, 4°C), and the supernatant was decanted. The washing step was repeated and then the cells stained in 25 μL with 5 μg/mL Penta-His-AF647 antibody at 4°C for 20 min. Finally, cells were again washed twice with ice-cold PBSA and the pelleted cells were resuspended in 60 μL ice-cold PBSA just before the measurement with a Cytoflex S instrument. Data were analyzed with FlowJo software. Cells were kept on ice to avoid endocytosis. *K*_D_ values were calculated by using a 1:1 binding model as described previously.^64^

#### Cross-competition assay

A431 and A549 cells were detached, washed and resuspended in ice-cold PBSA as described above. 10^5^ cells in 20 μL PBSA were transferred into a V-bottom 96-well plate and preincubated with 20 μL blocking reagent (13.5 μM ActE-binder without His_6_-tag, final concentration 4.5 μM) and 20 μL EGF (450 nM, final concentration 150 nM) at 4°C for 90 min. After the incubation time, 30 μL of detection reagent (180 nM His_6_-SUMO-tagged ActE-binder, final concentration 60 nM) was added, resulting in a final concentration of 3 μM for the blocking reagent and 100 nM for EGF, and further incubated at 4°C for 20 min. Subsequently, cells were washed twice as describe above and stained in 25 μL with 5 μg/mL Penta-His-AF647 antibody at 4°C for 20 min. Finally, cells were washed twice again with ice-cold PBSA and the pelleted cells were resuspended in 60 μL ice-cold PBSA just before the measurement with a Cytoflex S instrument. Data were analyzed with FlowJo software. Cells were kept on ice to avoid endocytosis.

#### EGFR signaling assay

The EGFR signaling assay was conducted as described by Wagner et al.^28^^,^^65^ Briefly, A431 cells were detached, washed and resuspended in ice-cold PBSA as described above. 2 × 10^5^ cells in 80 μL PBSA were used for each sample. Dilutions of His_6_-SUMO-tagged binders (4 μM) and EGF (30 nM and 150 nM) were prepared in PBSA. Either 10 μL His_6_-SUMO-tagged binder, 10 μL EGF or 20 μL of pre-made mixture of both were added resulting in a 1:10 dilution and a final concentration of 400 nM binder and 3 or 15 nM EGF, respectively. For samples with only His_6_-SUMO-tagged binder, with EGF only or for the negative control, PBSA was added to reach a total volume of 100 μL. After 5 min of incubation at 20°C, 1 mL of ice-cold methanol was added. To avoid clumping of the cells, cells were quickly vortexed after the addition of methanol. After 30 min incubation at 4°C, cells were washed twice with PBSA. For this, 3 mL of PBSA were added and the cells centrifuged (500 g, 5 min, 4°C). The supernatant was decanted, and the washing step repeated with 4 mL PBSA. After the second washing step, PBSA was added such that a defined volume of cell suspension (90 μL) could be transferred to a new tube to ensure a uniform staining volume for all samples. 10 μL of pEGFR (Tyr1068) antibody (clone D7A5, Cell Signaling Technology, 1:800 final dilution) was added to each tube and cells incubated at 20°C for 30 min in the dark. Next, the cells were washed again twice with PBSA and in the end 90 μL of cell suspension transferred into a new tube as described above. For secondary staining, 10 μL of anti-rabbit F(ab’)2 Fragment (AF647, Cell Signaling technology, 1:500 final dilution) was added and again incubated at 20°C for 30 min in the dark. After washing the cells again twice with PBSA, the cells in the remaining drop after decanting, were transferred into a 96-well plate and kept on ice before the measurement with a Cytoflex S instrument. Data were analyzed with FlowJo software.

#### Quantification of EGFR surface density

The surface densities of EGFR molecules on A431, A549 and SK-BR-3 cells were quantified using the QuantiBRITE Phycoerythrin (PE) Fluorescence Quantitation Kit (Becton Dickinson) according to the manufacturer's instructions. For determination of the surface expression upon ligand addition, 3 × 10^5^ cells in 0.5 mL culture medium were transferred into three reaction tubes (10 min time point) and in two 12-well plates, for each cell line, respectively. The ligand (EGF or TGF-α) was diluted in culture medium and the incubation started after the addition of 0.5 mL culture medium (control) or diluted ligand (final concentration 100 nM). For the 10 min time point, the reaction tubes were placed in the incubator and then directly on ice to avoid endocytosis. For the other time points, cells were first detached, followed by centrifugation (7 min, 300 g, 4°C), washing with 1 mL ice-cold PBSA, another round of centrifugation and finally resuspended in 150 μL PBSA. For each condition (without ligand or with EGF or TGF-α), ∼5 × 10^4^ cells in 25 μL were stained with anti-human EGFR antibody (PE, clone AY13; BioLegend, 1:50) in V-bottom 96-well plates at 4°C for 20 min. Subsequently, cells were washed twice, pelleted, and resuspended in 50 μL ice-cold PBSA just before the measurement with a Cytoflex S instrument. The gMFI was determined with FlowJo software, subjected to background subtraction using unstained cells and then used to estimate the number of antibodies bound per cell (ABC). ABC values were corrected for the PE-conjugation efficiency of the antibody (1.2 PE molecules/antibody) yielding effective surface densities. The cells were kept on ice to avoid endocytosis.

#### Nur77 reporter cell assay

Jurkat Nur77 reporter cells electroporated with CD19-BBζ CAR mRNA were co-cultured with Jurkat E6.1 target cells expressing CD19 (also through mRNA electroporation; both 16 h after electroporation) or with CD19^−^ Jurkat E6.1 cells at an E:T ratio of 1:2 (25,000:50,000 cells) at 37°C for 24 h. For co-culture experiments with EGFR-directed CARs, CAR expressing Nur77 cells (16 h after electroporation) were co-cultured with target cells at an Effector-to-Target (E:T) ratio of 1:1 (100,000 cells each) in the absence or presence of 100 nM of EGF or TGF-α at 37°C for 4 h, followed by analysis of the Nur77 activation level with a Cytoflex S instrument or LSR Fortessa (BD Life Science).

#### Cytokine secretion assay

CAR T cells (16 h after mRNA electroporation) were co-cultured with target cells at an E:T ratio of 1:1 (25,000 cells each) in the absence or presence of 100 nM EGF or TGF-α at 37°C for 4 h. Next, the plates were centrifuged (450 g, 7 min, 4°C) and the supernatant stored at −80°C. IFN-γ was analyzed using the ELISA MAX Deluxe Set Human IFN-γ (BioLegend) according to the manufacturer’s instructions. Analysis was performed with an Infinite 200 PRO (Tecan).

#### Cytotoxicity assay

CAR T cells (4 h after mRNA electroporation) were co-cultured with target cells (i.e., luciferase-expressing A431 and Raji) at an E:T ratio of 2.5:1 (25,000:10,000 cells) in white, round-bottom 96-well plates in the absence or presence of 100 nM EGF at 37°C for 4 h. The co-culture medium consisted of 150 μL RPMI-1640 (without phenol red) supplemented with 10% FBS superior, 1% penicillin-streptomycin solution and 200 U/mL IL-2. Before seeding, target cells were washed with RPMI (without phenol red) and the same culture medium as described above. 15 min before the end of the incubation time, the luciferin salt (IVISbrite D-Luciferin K+ Salt, PerkinElmer) was dissolved in sterile water to create a 150x stock solution (22.5 mg/mL), which was further diluted in the same culture medium to achieve a 4x concentration. After the incubation time, 50 μL of 4x Luciferin stock solution was added to each well (final concentration 150 μg/mL) and the plate incubated at 20°C for 20 min in the dark. The luminescence was measured with a Victor X5 (PerkinElmer). Lysis (%) normalized to target cells was determined with the following formula (RLU, relative light units):

$$\text{Lysis}\;\left(\%\right)=100-\;\frac{\text{RLU}\;\text{from}\;\text{well}\;\text{with}\;\text{effector}\;\text{and}\;\text{target}\;\text{cell}\;\text{co}-\text{culture}}{\text{RLU}\;\text{from}\;\text{well}\;\text{with}\;\text{target}\;\text{cells}\;\text{only}}\;x\;100$$

### Quantification and statistical analysis

Statistical analysis was performed using GraphPad Prism v10.1.1 software for Windows (GraphPad Software). Data are presented as individual data points or as means ± SD. Statistical analysis was done by two-way ANOVA and different multiple comparison tests. In Figures 4A, 7C, and S5, Sidak’s multiple comparison tests were used, in Figures 6B and 7B Tukey post-hoc test, and Figure S8 Dunnett’s multiple comparison.

## Supplemental information

Document S1. Figures S1‒S7

Document S2. Article plus supplemental information
