# Supplementary material for: An engineering strategy to target activated EGFR with CAR T cells
Source: Cell Rep Methods. 2024 Mar 15;4(4):100728. doi: 10.1016/j.crmeth.2024.100728 (PMC11045874; doi:10.1016/j.crmeth.2024.100728)
Supplement: Document S1. Figures S1‒S7 [file mmc1.pdf]

Cell Reports Methods, Volume *4*

Supplemental information

An engineering strategy to target activated EGFR with CAR T cells

Markus Dobersberger, Delia Sumesgutner, Charlotte U. Zajc, Benjamin Salzer, Elisabeth Laurent, Dominik Emminger, Elise Sylvander, Elisabeth Lehner, Magdalena Teufl, Jacqueline Seigner, Madhusudhan Reddy Bobbili, Renate Kunert, Manfred Lehner, and Michael W. Traxlmayr

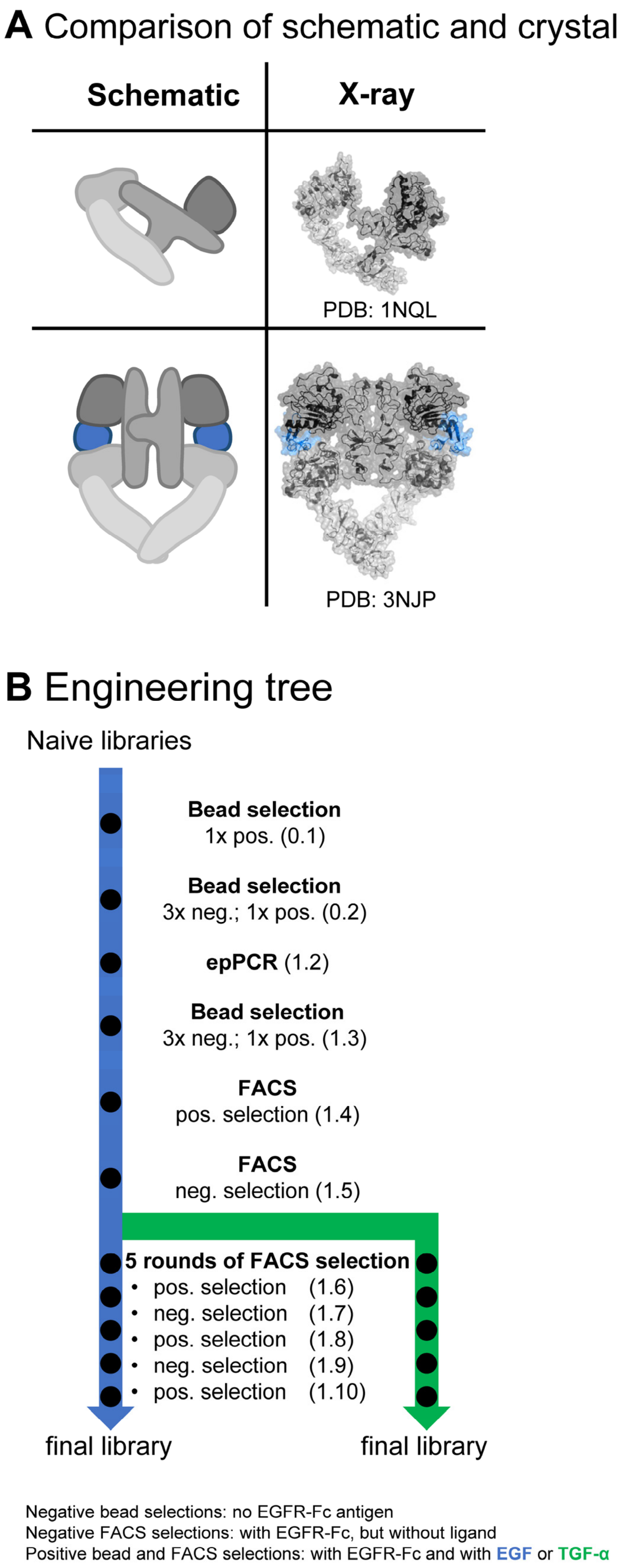
A Comparison of schematic and crystal structure of EGFR
Schematic
X-ray
PDB: 1NQL
PDB: 3NJP
B Engineering tree
Naive libraries
Bead selection
1x pos. (0.1)
Bead selection
3x neg.; 1x pos. (0.2)
epPCR (1.2)
Bead selection
3x neg.; 1x pos. (1.3)
FACS
pos. selection (1.4)
FACS
neg. selection (1.5)
5 rounds of FACS selection
• pos. selection (1.6)
• neg. selection (1.7)
• pos. selection (1.8)
• neg. selection (1.9)
• pos. selection (1.10)
final library
final library
Negative bead selections: no EGFR-Fc antigen
Negative FACS selections: with EGFR-Fc, but without ligand
Positive bead and FACS selections: with EGFR-Fc and with EGF or TGF-α

Figure S1. Structural change of the EGFR extracellular domain and library selection strategy, related to Figures 1 and 2.

(A) Schematic representations of monomeric and dimeric EGFR, as well as structures obtained by X-ray crystallography (PDB-IDs 1NQL[1] and 3NJP[2] for tethered, monomeric and EGF-bound, dimeric EGFR, respectively). Different gray colors represent the four domains of the extracellular part of EGFR. Dimeric EGFR is bound to EGF shown in blue. **(B)** Yeast surface display engineering tree starting from the naïve libraries rcSso7d-11 and rcSso7d-18 developed by Traxlmayr et al.[3] In positive selections (pos.), binders were enriched which bind to soluble EGFR-Fc in the presence of a ligand (EGF or TGF-α, respectively). In negative bead selections, binders not interacting with bare beads (i.e. in the absence of EGFR-Fc) were selected, whereas in negative FACS selections, binders not binding to EGFR-Fc in the absence of ligands were enriched. In the first five rounds EGF (blue) was used for the positive selections and then the library was split into two arms – one with EGF and the other with TGF-α (green) for positive selections. Numbers in brackets indicate the names of the respective libraries.

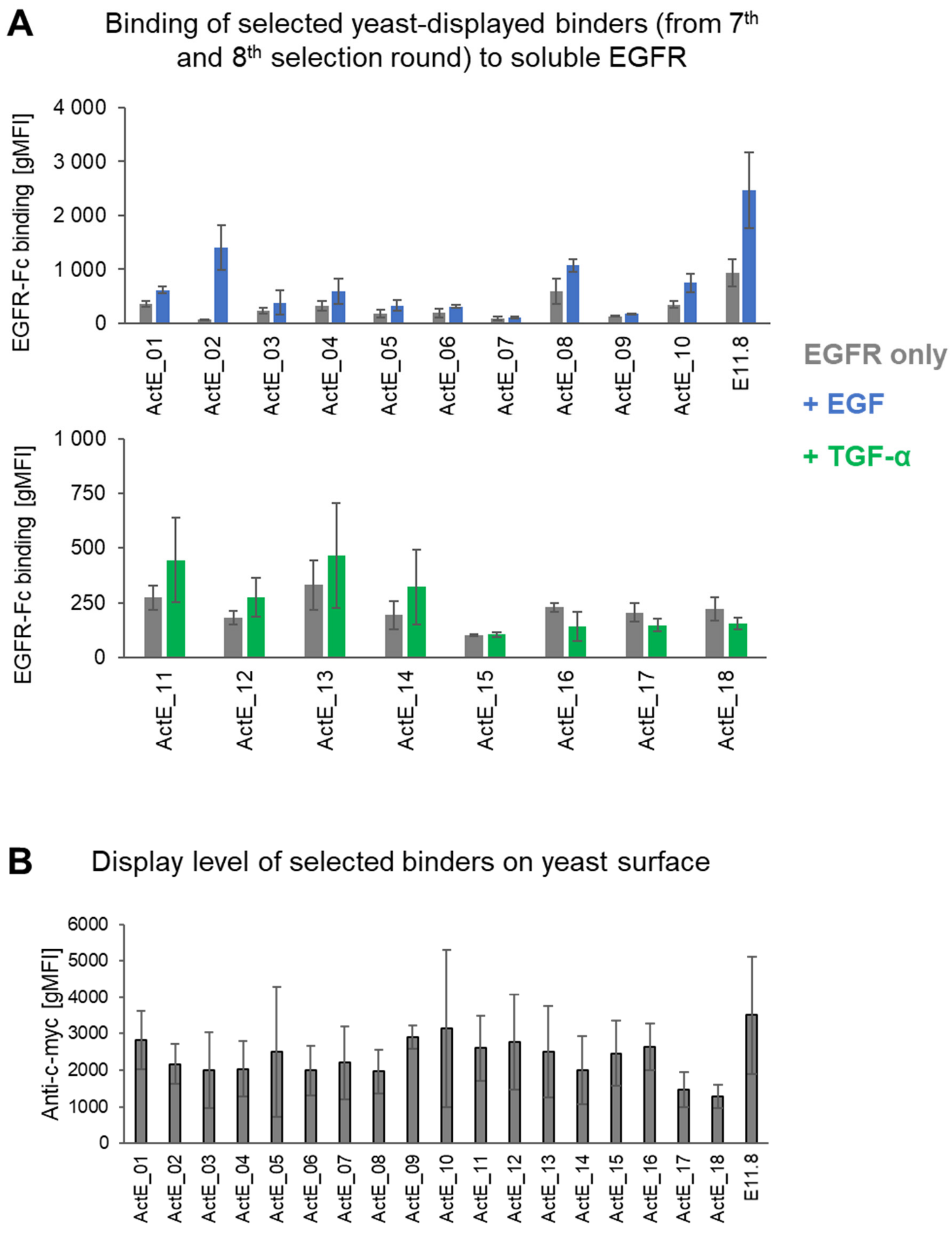

Figure S2. Testing of individual binders isolated after the 7th and 8th selection round, related to Figure 2. **(A)** Binders obtained after the 7th or 8th selection round were displayed on yeast and tested for binding (geometric mean fluorescence intensity, gMFI) to 15 nM soluble EGFR-Fc in the absence (gray) and presence of 100 nM of the EGFR-ligand they were sorted against (EGF blue; TGF-α green). **(B)** Flow cytometric analysis of display levels (i.e. surface expression levels) of selected binders on yeast cells by using an antibody recognizing the C-terminally expressed c-myc tag (mean ± SD of three independent experiments). All gMFI values were background-subtracted.

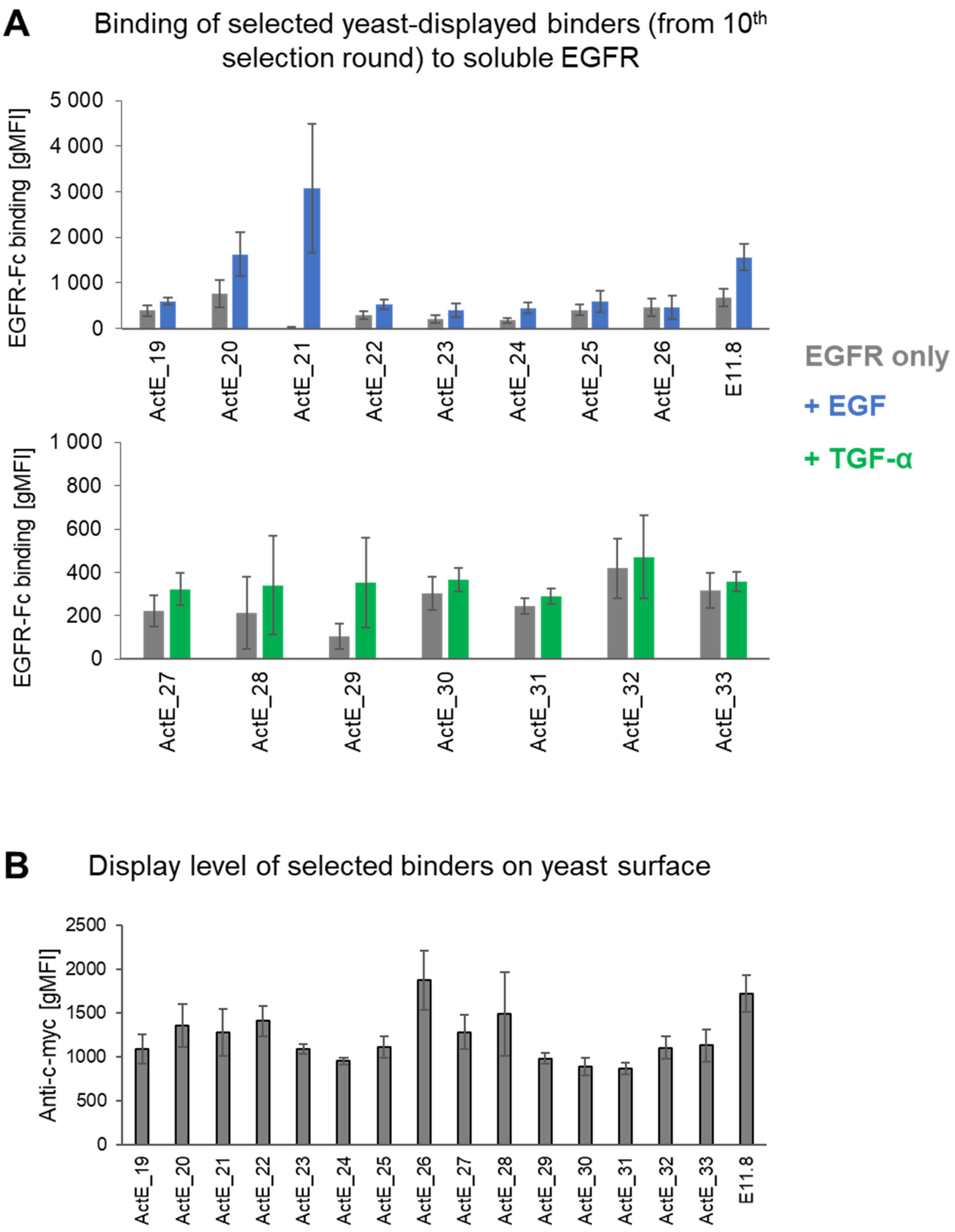

Figure S3. Testing of individual binders isolated after the 10th selection round, related to Figure 2.
(A) Binders obtained after the 10th selection round were displayed on yeast and tested for binding (geometric mean fluorescence intensity, gMFI) to 15 nM soluble EGFR-Fc in the absence (gray) and presence of 100 nM of the EGFR-ligand they were sorted against (EGF blue; TGF-α green). **(B)** Flow cytometric analysis of display levels (i.e. surface expression levels) of selected binders on yeast cells by using an antibody recognizing the C-terminally expressed c-myc tag (mean ± SD of three independent experiments). All gMFI values were background-subtracted.

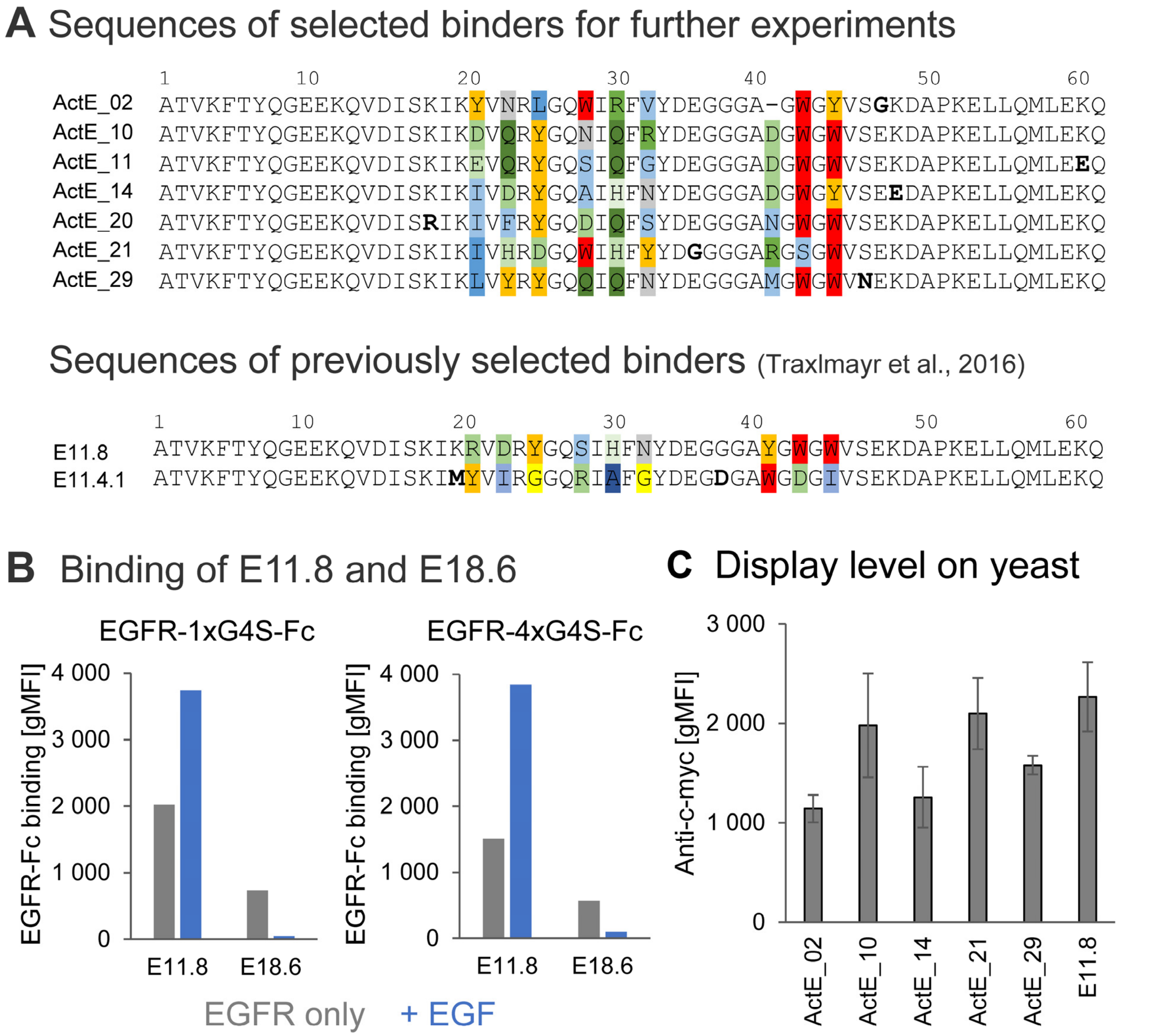

Figure S4. Sequence of selected binders, binding of soluble EGFR-Fc to E11.8 and E18.6 and display levels on yeast, related to Figures 2 and 3.

(A) Sequences of selected ActE variants, as well as binders included from a previous study[3] are shown. Identical amino acids within the nine positions of the engineered binding interface are depicted in the same colors. Framework mutations are highlighted in bold, and deletions with a hyphen. **(B)** Binding of E11.8 and E18.6 expressed on yeast cells to 25 nM soluble EGFR-Fc with different linker lengths (1xG4S and 4xG4S, respectively) as indicated in the absence (gray) or presence of 50 nM EGF (blue). EGFR-Fc binding (geometric mean fluorescence intensity, gMFI) was analyzed by flow cytometry. With each EGFR-Fc antigen (containing a 1xG4S or 4xG4S linker, respectively) one experiment was performed, yielding similar results independent of the type of linker. **(C)** Flow cytometric analysis of display levels (i.e. surface expression levels) of selected binders on yeast cells by using an antibody recognizing the C-terminally expressed c-myc tag (mean ± SD of three independent experiments). All gMFI values were background-subtracted.

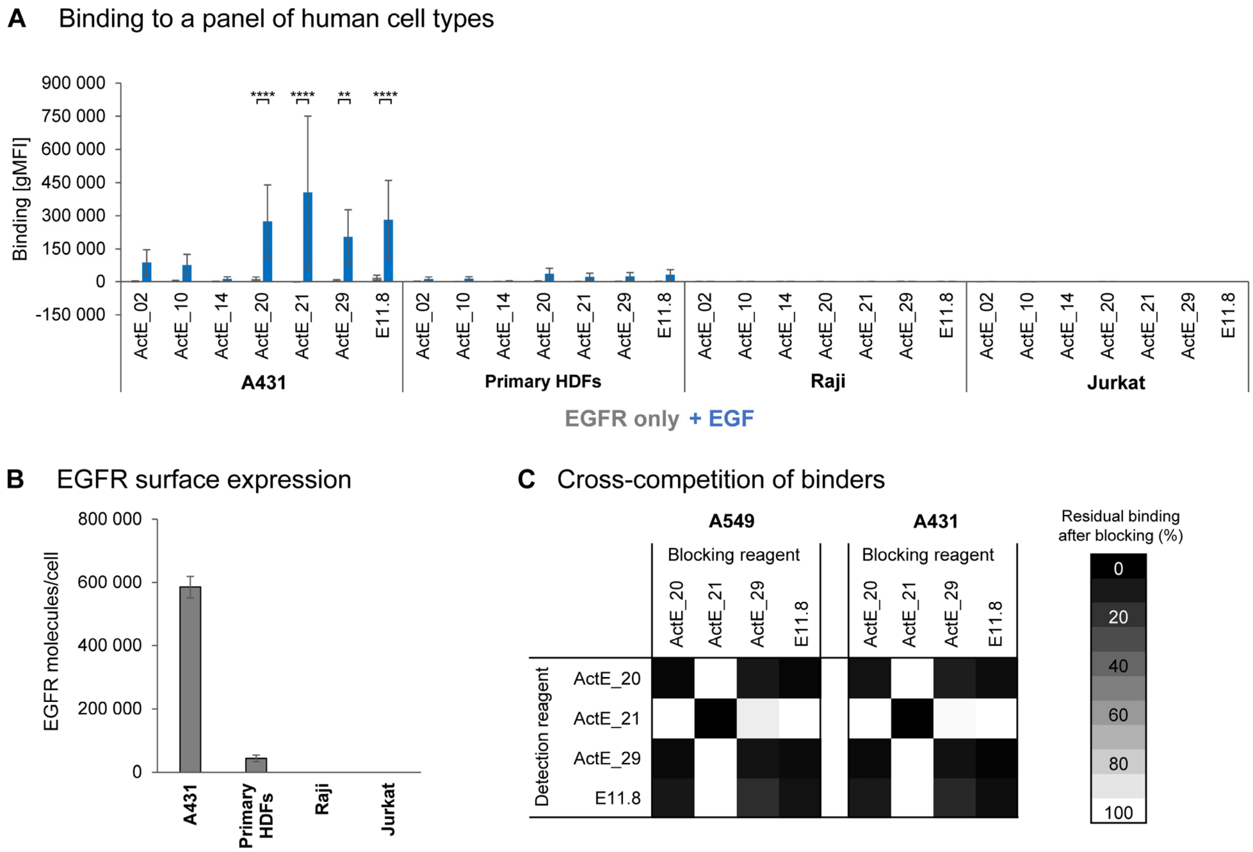

Figure S5. Binding to a panel of human cell types, EGFR surface expression on this cell panel and cross-competition of binders, related to Figure 4.

(A) Different human cell types were incubated with engineered binders (100 nM, expressed as SUMO fusion proteins) in the absence (gray) or presence of 100 nM EGF (blue), followed by flow cytometric analysis of bound binders (geometric mean fluorescence intensity, gMFI). Mean ± SD of three independent experiments. Statistical significance was calculated via two-way ANOVA and Sidak's multiple comparisons test (**** = $p<0.0001$, ** = $p<0.01$). **(B)** EGFR levels on the surface of A431, primary human dermal fibroblasts (HDFs), Raji and Jurkat cells measured by flow cytometry and quantification beads. Mean ± SD of three independent experiments. **(C)** A431 and A549 cells were preincubated with engineered binders (4.5 μM) without His_6-tag (blocking reagent) in the presence of 150 nM EGF, followed by the addition of 180 nM His_6-SUMO-tagged binder (detection reagent). Subsequently, binding was analyzed by flow cytometry. Average blocking levels of three independent experiments are shown. All gMFI values were background-subtracted.

A Activation of a CD19-BBζ CAR in Jurkat Nur77 reporter cells

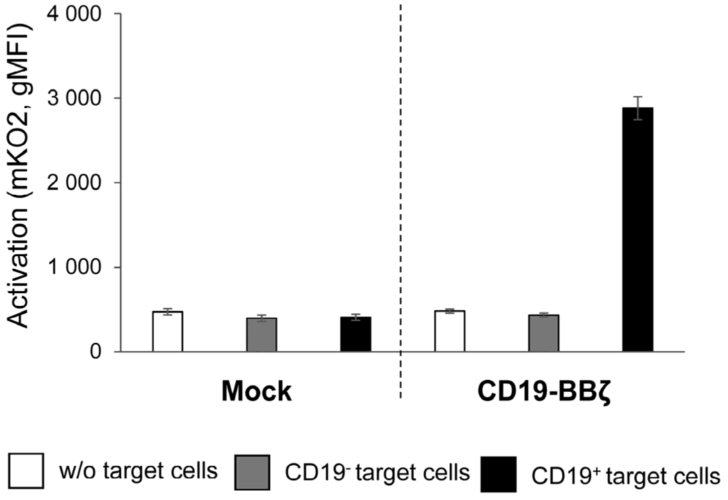

B CAR Expression in Jurkat Nur77 reporter cells

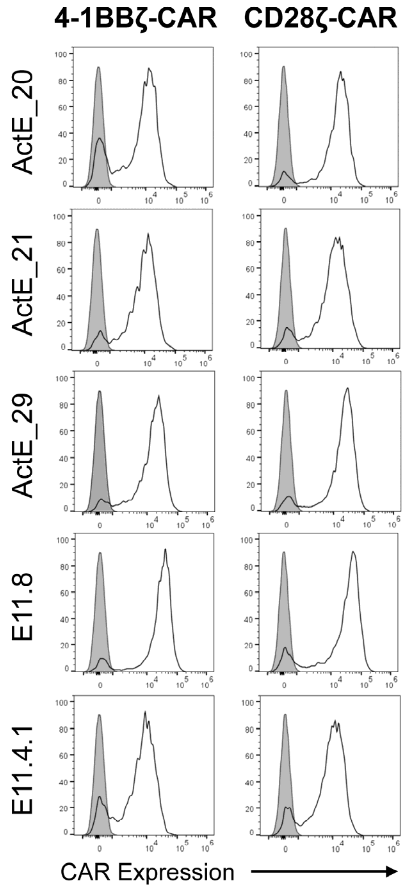

C CD4/CD8 ratio of primary T cells

Donor	IFN-γ secretion experiment		
	I	II	III
B1	40/50	35/57	35/57
B3	49/47	52/44	44/51

D Sequences of CAR construct and domains

**scFv (including linker):**	
FMC63 - CD19 scFv	DIQMTQTTSSLSASLGDRVTISCRASQDISKYLNWYQQKPDGTVKLLIYHTSRLHS GVPSRFSGSGSGTDYSLTISNLEQEDIATYFCQQGNTLPYTFGGGTKLEITGSTSG SGKPGSGEGSTKGEVKLQESGPGLVAPSQSLSVTCTVSGVSLPDYGVSWIRQPP RKGLEWLGVIWGSETTYYNSALKSRLTIIKDNSKSQVFLKMNSLQTDDTAIYYCAKH YYYGGSYAMDYWGQGTSVTVSS
**Hinge domains:**	
CD28 Hinge	IEVMYPPPYLDNEKSNGTIIHVKGKHLCPSPLFPGPSKP
CD8 Hinge	TTTPAPRPPTPAPTIASQPLSLRPEACRPAAGGAVHTRGLDFACD
**Transmembrane domains:**	
CD28 Transmembrane	FWVLVVVGGVLACYSLLVTVAFIIFWV
CD8 Transmembrane	IYIWAPLAGTCGVLLLSLVITLYC
**Costimulatory domains:**	
CD28	RSKRSRLLHSDYMNMTPRRPGPTRKHYQPYAPPRDFAAYRS
4-1BB	KRGRKKLLYIFKQPFMRPVQTTQEEDGCSCRFPEEEEGGCEL
**CD3ζ domains:**	
CD3ζ	RVKFSRSADAPAYQQGQNQLYNELNLGRREEYDVLDKRRGRDPEMGGKPRRKN PQEGLYNELQKDKMAEAYSEIGMKGERRRGKGHDGLYQGLSTATKDTYDALHMQ ALPPR
CD3ζ Q65K (CD19-CAR)	RVKFSRSADAPAY**K**QGQNQLYNELNLGRREEYDVLDKRRGRDPEMGGKPRRKN PQEGLYNELQKDKMAEAYSEIGMKGERRRGKGHDGLYQGLSTATKDTYDALHMQ ALPPR

Figure S6. Activation of CD19-BBζ CAR in Jurkat Nur77 reporter cells, CAR Expression in Jurkat Nur77 reporter cells, CD4/CD8 ratio of primary T cells and sequences of CAR domains, related to Figures 5, 6 and 7. **(A)** Geometric mean fluorescence intensity (gMFI) of the activation level (mKO2) of CD19-BBζ CAR or Mock (no CAR) Jurkat Nur77 reporter cells either alone or co-cultured with $CD19^-$ or $CD19^+$ target cells (mean ± SD of three independent experiments). **(B)** Flow cytometric analysis of CAR expression in Jurkat Nur77 reporter cells for the indicated constructs. **(C)** CD4/CD8 ratio of T cells of the indicated donors on the day of the IFN-γ secretion experiments. **(D)** Sequences of the different CAR domains.

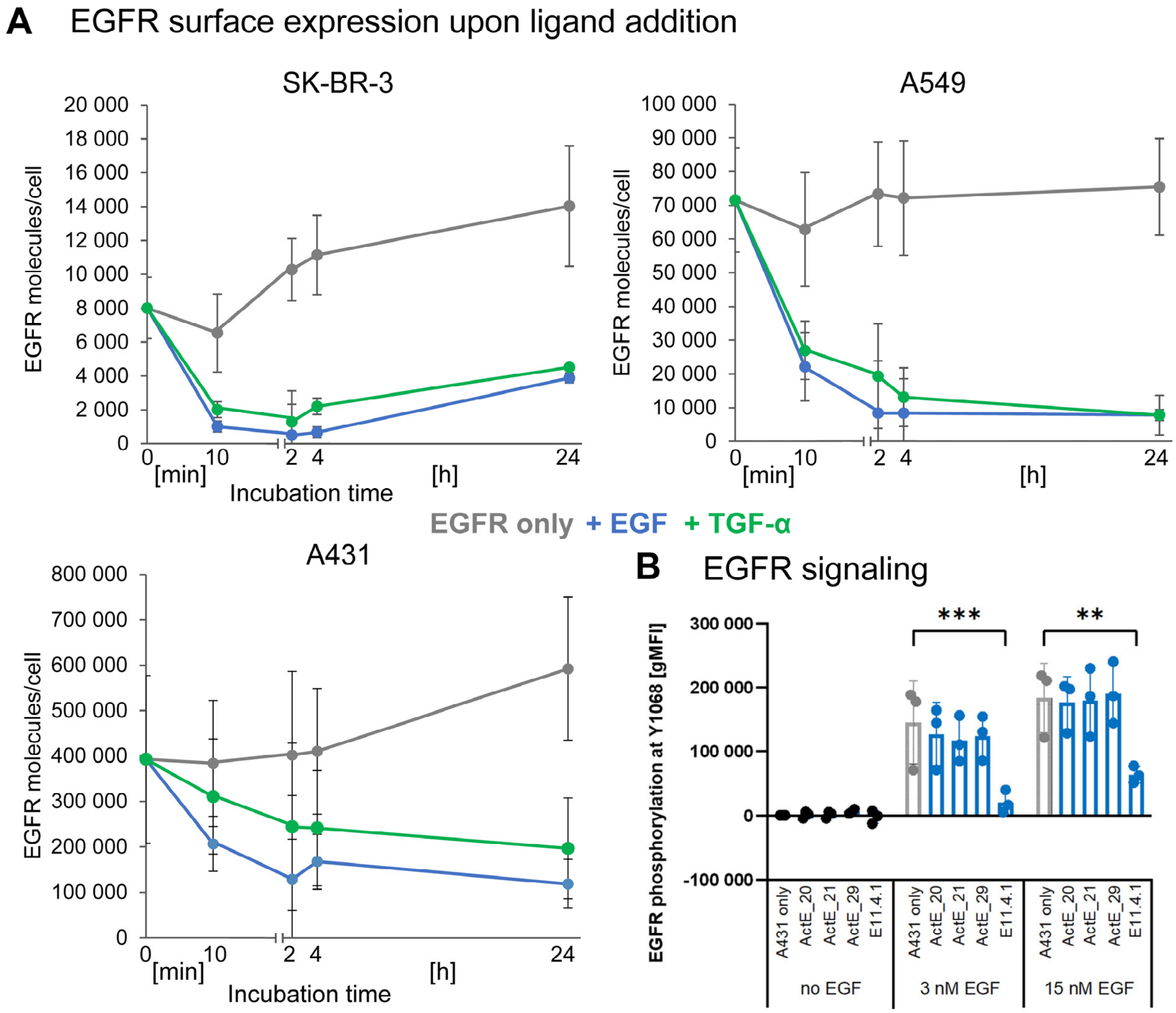

Figure S7. EGFR surface expression upon addition of ligands and EGFR signaling, related to Figures 6 and 7. **(A)** Number of EGFR molecules on the surface of the indicated target cell lines (A431, A549 and SK-BR-3) either in the absence of EGFR ligands (gray) or after incubation with EGF (blue) or TGF-α (green) at the indicated time points measured by flow cytometry and quantification beads. **(B)** A431 cells were incubated with engineered binders (400 nM) in the absence or presence of EGF (3 or 15 nM) for 5 minutes, followed by fixation and permeabilization with methanol. EGFR phosphorylation (geometric mean fluorescence intensity, gMFI) at Tyr1068 was determined by flow cytometry (mean ± SD of three independent experiments). Statistical significance was calculated via two-way ANOVA and Dunnett's multiple comparisons test (*** = $p<0.001$, ** = $p<0.01$).

Supplemental references

1. Ferguson, K.M., Berger, M.B., Mendrola, J.M., Cho, H.S., Leahy, D.J., and Lemmon, M.A. (2003). EGF activates its receptor by removing interactions that autoinhibit ectodomain dimerization. Mol Cell *11*, 507-517.
2. Lu, C., Mi, L.Z., Grey, M.J., Zhu, J., Graef, E., Yokoyama, S., and Springer, T.A. (2010). Structural evidence for loose linkage between ligand binding and kinase activation in the epidermal growth factor receptor. Mol Cell Biol *30*, 5432-5443. 10.1128/MCB.00742-10.
3. Traxlmayr, M.W., Kiefer, J.D., Srinivas, R.R., Lobner, E., Tisdale, A.W., Mehta, N.K., Yang, N.J., Tidor, B., and Wittrup, K.D. (2016). Strong Enrichment of Aromatic Residues in Binding Sites from a Charge-neutralized Hyperthermostable Sso7d Scaffold Library. J Biol Chem *291*, 22496-22508. 10.1074/jbc.M116.741314.
